# Clinical applications of mesenchymal stromal cell-based therapies for pulmonary diseases: An Update and Concise Review

**DOI:** 10.7150/ijms.59218

**Published:** 2021-06-01

**Authors:** Xiaobo Chen, Feng Wang, Zhiwei Huang, Yan Wu, Jie Geng, Yuliang Wang

**Affiliations:** 1 Unicell Life Science Development Co., Ltd, Tianjin, China.; 2 Department of Genetics, School of Basic Medical Sciences, Tianjin Medical University, Tianjin, China.; 3 Department of Clinical Laboratory Medicine, the Second Hospital of Tianjin Medical University, Tianjin Institute of Urology, Tianjin, China.; 4 Department of Clinical Laboratory Medicine, Tianjin TEDA Hospital, Tianjin, China.

## Abstract

Lung disorders are a leading cause of morbidity and death worldwide. For many disease conditions, no effective and curative treatment options are available. Mesenchymal stromal cell (MSC)-based therapy is one of the cutting-edge topics in medical research today. It offers a novel and promising therapeutic option for various acute and chronic lung diseases due to its potent and broad-ranging immunomodulatory activities, bacterial clearance, tissue regeneration, and proangiogenic and antifibrotic properties, which rely on both cell-to-cell contact and paracrine mechanisms. This review covers the sources and therapeutic potential of MSCs. In particular, a total of 110 MSC-based clinical applications, either completed clinical trials with safety and early efficacy results reported or ongoing worldwide clinical trials of pulmonary diseases, are systematically summarized following preferred reporting items for systematic reviews and meta-analyses (PRISMA) guidelines, including acute/viral pulmonary disease, community-acquired pneumonia (CAP), chronic obstructive pulmonary disease (COPD), bronchopulmonary dysplasia (BPD), interstitial lung diseases (ILD), chronic pulmonary fibrosis, bronchiolitis obliterans syndrome (BOS) and lung cancer. The results of recent clinical studies suggest that MSCs are a promising therapeutic approach for the treatment of lung diseases. Nevertheless, large-scale clinical trials and evaluation of long-term effects are necessary in further studies.

## Introduction

Lung diseases across all ages have become one of the major public health issues worldwide with increased human activities, environmental changes, indoor and outdoor air pollution, long-term smoking, occupational exposures, and various pathogens 1. Acute and chronic lung diseases have high morbidity and mortality 2,3. While these diverse conditions require different specific therapeutic approaches (e.g., antimicrobial medications, inhaled corticosteroids, anti-inflammatory drugs, antifibrotic drugs, specific cytokine inhibitors, bronchodilators, respiratory support, mechanical ventilation, and restricted fluid input), persistent alleviation of clinical symptoms cannot be offered to most patients affected to date. Lung transplantation has evolved to represent the last option for many patients with end-stage lung diseases. However, there is a severe shortage of suitable donor lungs, and transplantation itself is associated with the potential for serious risks due to the need for lifelong immunosuppression, resulting in a high posttransplant mortality rate 4. Thus, a new therapeutic strategy is desperately needed.

Mesenchymal stromal cell (MSC)-based therapy is one of the most cutting-edge and popular directions in medical research today 5. Autologous or allogeneic-derived mesenchymal stem cells (MSCs) are easier to obtain from multiple biological tissues, including bone marrow (BM), neonatal tissues, and adipose tissues. MSCs can be induced into proinflammatory MSC type 1 (MSC1) or anti-inflammatory MSC type 2 (MSC2) responding to different immune environments 6. These cells have multiple potential advantages, including superior proliferation ability, lower immunogenicity, multidifferentiation potential, large-scale supply, and minimal ethical issues 7. Upon administration by the intravenous route, the cells travel directly to the lungs, where the majority are sequestered, a great benefit for the treatment of pulmonary disease. These findings have paved the way for the development of clinical protocols and thereby provide off-the-shelf therapy.

To globally analyze clinical trials for MSC-based therapy of pulmonary diseases, a comprehensive search of the ClinicalTrials.gov database from 1990 to January 19, 2021, was conducted according to PRISMA guidelines. We systematically summarized completed and ongoing clinical trials worldwide of pulmonary diseases, including acute/viral pulmonary disease, community-acquired pneumonia (CAP), chronic obstructive pulmonary disease (COPD), bronchopulmonary dysplasia (BPD), interstitial lung diseases (ILD), chronic pulmonary fibrosis, bronchiolitis obliterans syndrome (BOS) and lung cancer. Moreover, the sources and therapeutic potential of MSCs are also summarized. The results of current clinical studies support MSCs as a promising therapeutic approach for the treatment of lung diseases. Nevertheless, large-scale clinical trials and evaluation of long-term effects are necessary in further studies.

## Adult and Neonatal Tissue Source of MSCs

MSCs are nonhematopoietic stem cells with multilineage potential and can be readily isolated and expanded from multiple biological tissues, including BM, neonatal tissues, and adipose tissues. MSCs are an attractive stem cell source for the regeneration of damaged tissues in clinical applications because these cells are characterized as undifferentiated cells, are able to self-renew with a high proliferative capacity, and possess mesodermal differentiation potential 8. MSCs can not only modulate immune responses in different inflammatory microenvironments but also relieve cell death and tissue injury in pathological and physiological states 9. MSCs can be extracted from both healthy donors and patients and are easily expanded *in vitro* to a therapeutic volume used as an “off-the shelf” therapeutic agent or can be stored for repetitive therapeutic usage 10. BM is the most characterized and documented source of MSCs. BM-derived MSCs (BMSCs) have become the most common source of multipotent cells for transplantation in preclinical and clinical trials since they were first isolated in 1970 by Friedenstein et al. 11. However, the harvest of MSCs from BM is a painful, invasive procedure, and there is a risk of viral exposure. In addition, the number, differentiation potential, and maximal life span of MSCs from BM decline with increasing age 12. MSCs in the umbilical cord (UC) can be obtained from Wharton jelly, veins, arteries, the umbilical cord lining, and the subamnion and perivascular regions. UC-derived MSCs (UCMSCs) can be obtained through a painless collection method and have fewer associated ethical issues. They also renew faster than BMSCs 13. Adipose tissues are another popular source and have significant advantages over MSCs derived from other sources, mainly because a large number of MSCs can be obtained through minimally invasive lipoaspiration methods and can easily be extracted 14. The MSC concentration in adipose tissue is greater than that in all other tissues in the body, and the potency is maintained with the age of the donor, unlike BMSCs. In addition, adipose-derived MSCs (ADSCs) possess stronger immunomodulatory capability than BM-MSCs 15. Taken together, these findings show that ADSCs have advantages in both autologous use and allogeneic use. Since the mid-2000s, thousands of clinical trials have used MSCs to test therapeutic interventions for numerous severe diseases, alone or in combination with other drugs. Notably, as a paracrine mediator, exosome-based therapy is now recognized as an emerging novel approach that contributes to the healing of injured or diseased tissues and organs 16. Exosomes (50-150 nm in diameter) derived from MSCs may contain growth factors, cytokines, DNA, lipids, mRNAs, miRNAs, and mtRNAs, which function as intercellular mediators between MSCs and target cells, including MSCs. MSC-derived exosomes possess therapeutic properties, including stimulation of cell migration and extracellular matrix synthesis, antiapoptotic effects, immunomodulation and anti-inflammatory effects 17.

## Therapeutic Potential of MSCs

**Figure 1:**
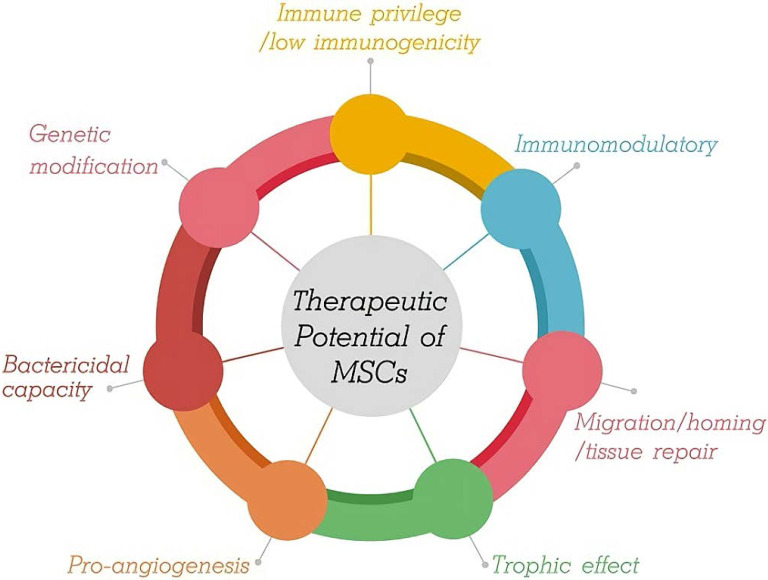
Therapeutic Potential of MSCs.

During the last decade, rapidly developing regenerative medicine in the treatment of tissue and organ injury has led to more widespread use of MSC technology.* In vitro*, MSCs show self-renewal, extensive proliferation ability, and multipotency. The therapeutic potential of MSCs for lung disorders is supported by several factors (Figure 1).

First, MSCs are characterized by low immunogenicity. Generally, MSCs positively express CD73, CD90, and CD105 surface markers, have low expression of major histocompatibility complex (MHC) class I, and do not express hematopoietic or endothelial surface markers (CD11b, CD14, CD19, CD34, CD45, CD79alpha), human leukocyte antigen (HLA)-DR, major histocompatibility complex (MHC) class II, costimulatory molecules (e.g., CD40/CD40L, B7/CD28, ICOS/ICOSL, 4-1BB/4-1BBL, OX40/OX40L), or adhesion molecules (e.g., CD31, CD18, and CD56) 18; additionally, transmembrane 4 L6 family member 1 (TM4SF1) has been indicated effective as an MSC-specific surface marker 19. Therefore, this low-immunogenicity phenotype of MSCs permits the use of allogeneic cells for patients and strongly reduces the risk of allograft rejection. No immunosuppressive therapy is needed.

**Figure 2:**
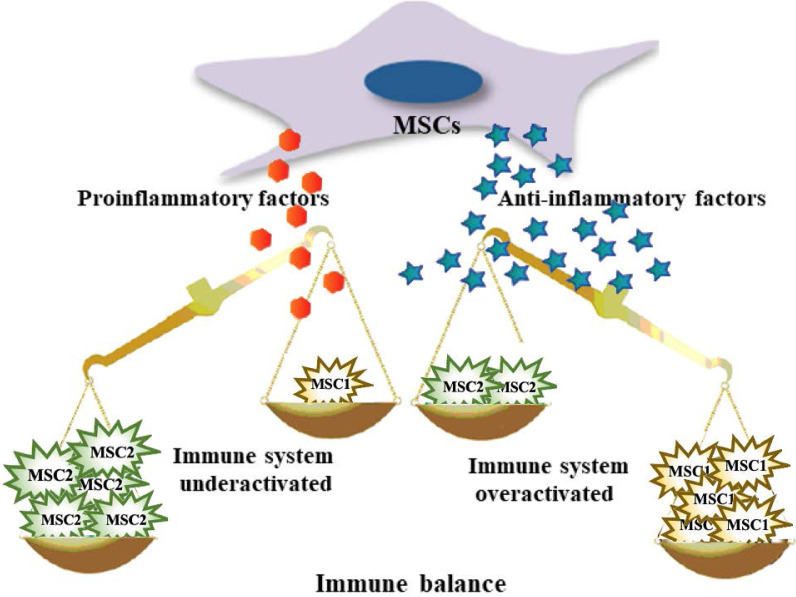
MSCs exhibit both anti-inflammatory and pro-inflammatory effects.

Second, MSCs modulate the host immune response. The role of MSCs is to adjust the balance between inflammation and tissue reconstruction to provide damaged tissue with a relatively stable environment, which is beneficial for tissue repair. MSCs from the microenvironment are considered to constitute a double-edged sword in exerting multiple modulatory effects on diverse aspects of the immune response. That is, MSCs are capable of polarized differentiation 20. MSCs can differentiate into MSC1 cells, which can promote a proinflammatory state and preserve the immune response to microorganisms through specific Toll-like receptors (TLRs) when the immune system is underactivated 21. On the other hand, when the immune system is overactivated (cytokine storm), MSCs may differentiate into anti-inflammatory MSC2 cells to limit the inflammatory cytokine cascade and host tissue injury, ultimately avoiding self-overattack (Figure 2) 22,23. More specifically, MSCs can be used as therapy to strike a balance in the immune cells of patients with COVID-19. It has been proposed that MSCs suppress cytokine storms by negatively regulating the immune response in the case of major inflammation (as with COVID-19) 24.

Third, MSCs enhance migration/homing and tissue repair after injury, which is mediated partly by paracrine and/or directed differentiation mechanisms that enhance the resolution of tissue injury. After sensing the injury signal released from damaged tissues, MSCs can be mobilized and migrate into injured tissues through peripheral circulation; this trafficking process is regulated by multiple mechanical factors (e.g., mechanical strain, shear stress, matrix stiffness, and microgravity) and chemical factors (including stromal derived factor-1/CXC chemokine receptor 4 axis, osteopontin, basic fibroblast growth factor, vascular endothelial growth factor-A, hepatocyte growth factor, insulin-like growth factor-1, platelet-derived growth factor, transforming growth factor-β1) 25. Subsequently, MSCs reach the damaged tissue site and perform wound healing of damaged tissues in two key ways, i.e., paracrine (e.g., releasing bioactive factors: chemokines, cytokines, and growth factors) and/or directed differentiation to replace damaged cells (e.g., osteocytes, chondrocytes, cardiomyocytes, and endothelial cell differentiation) 26. Over the recent decades of intensive studies, the bone morphogenic protein (BMP) signaling and wingless and int-1 (Wnt) signaling pathways have been demonstrated to regulate osteoblast and adipocyte differentiation of MSCs 27. The sustained activation of ERK by 5-azacytidine contributed to the induction of the differentiation of MSCs into cardiomyocytes 28. Growth differentiation factor 11 (GDF11) binds to the TGF-β receptor and subsequently activates the RAS-RAF-MEK-ERK/EIF4E pathway to induce the endothelial differentiation of MSCs 29. A body of evidence indicates that following systemic injection, most MSCs are trapped in capillary beds of various tissues, especially the lungs. MSC infusion might benefit alveolar epithelial cells, injured airways and lung tissue repair given the ability of these cells to differentiate into targeted cells to counteract pulmonary fibrosis and improve lung dysfunction.

Fourth, MSCs enhance trophic effects. The trophic properties of MSCs are believed to be a mechanism underpinning the therapeutic impact in preclinical studies. MSCs can either promote their own survival and proliferation through autocrine effects or secrete trophic factors that will act on adjacent cells through a paracrine effect in a hostile microenvironment 30. For example, prostaglandin E2 secreted by MSCs contributes to the maintenance of self-renewal capacity through the E-prostanoid 2 receptor 31.

Fifth, MSCs induce pro-angiogenic properties. Angiogenesis is a complex biological process involving interactions between vascular cells and the extracellular environment, and its dysregulation can contribute to serious disease. A growing body of evidence has shown that MSC-based proangiogenic therapies have been increasingly utilized in the treatment of ischemic diseases 32. This effect was mainly attributed to the modulation of angiogenic factors produced by MSCs. Roura et al. reported that umbilical cord blood-derived MSCs showed angiogenic potential since they directly self-organize, forming new functional vasculature connected with the host circulatory system once implanted 33. Recent experimental studies have demonstrated that MSC-derived exosomes could be considered for use in therapeutic angiogenesis, especially for ischemic diseases 34. More interestingly, miR29a-loaded exosomes from engineered BMSCs (miR-29a-loaded BMSC-Exos) showed a robust ability to promote angiogenesis and osteogenesis *in vivo* 35.

Sixth, MSCs may enhance host antimicrobial capacity. MSCs have demonstrated bactericidal effects both in vitro and in vivo through direct and indirect mechanisms to induce microbial killing. Direct mechanisms of MSC-mediated bacterial killing include scavenger receptor-mediated phagocytosis (macrophage receptor with collagenous structure (MARCO) and SR-B1), antimicrobial peptide (AMP) production, and the indoleamine 2,3-dioxygenase (IDO) and inducible nitric oxide synthase (iNOS) pathways 36. Recent evidence has suggested that MSCs have the potential to break down biofilms via cysteine protease secretion and present a strategy to increase the efficacy of conventional antibiotics via combination therapy between degradation of the biofilm layer by MSCs and increased antibiotic penetration 37,38. Indirect mechanisms of action are through the recruitment and activation of host immune cells. MSC administration can result in enhanced alveolar macrophage phagocytosis involved in promoting effective antigen presentation, phagocytosis, and bacterial killing. MSC-derived extracellular vesicles (EVs) carrying mitochondria are responsible for these effects through the promotion of oxidative phosphorylation in macrophages 39,40. In addition, in an *in vitro* virus infection experiment, MSCs demonstrated antiviral effects and could inhibit virus-specific CD8 (+) T-cell proliferation activation and proliferation via IDO-mediated mechanisms 41. Literature reviews demonstrate that specific TLR stimulation affects the immunomodulatory potency of MSCs. Given that TLRs are immediately capable of detecting internal and external hazard signals and that their stimulation has an intense effect on the ability to proliferate, differentiate, migrate, and survive, it seems that stimulation of these receptors can have a primary effect on the interaction of MSCs and immune cells, improving the antiviral activity 42.

**Figure 3:**
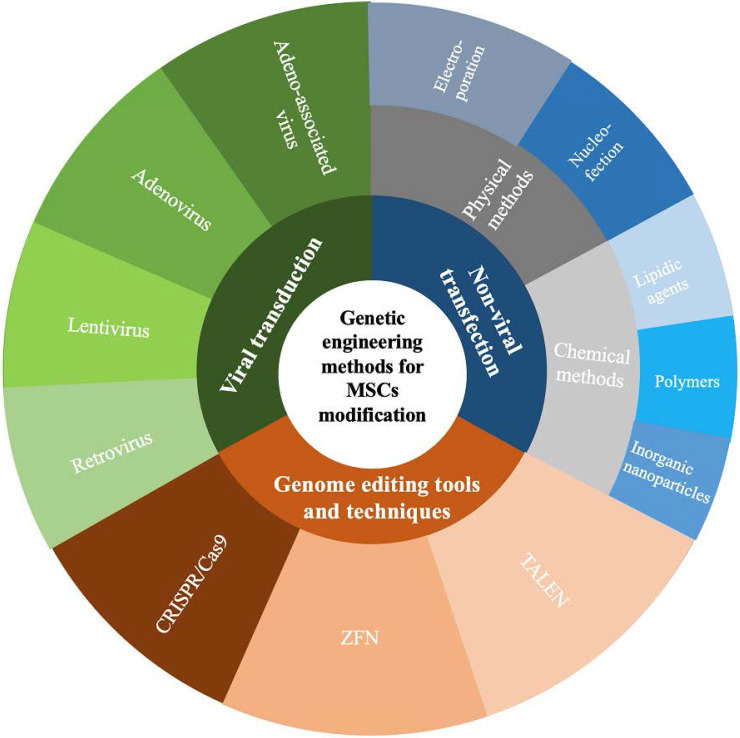
Genetic engineering methods for MSCs modification. CRISPR/Cas9, clustered regularly interspaced short palindromic repeats/CRISPR-associated 9; ZFN, zinc finger nuclease; TALEN, transcription activator-like effector nucleases.

Seventh, genetic engineering strategies represent a promising and effective approach to enhance the therapeutic efficacy of MSCs and improve the outcomes of diseases. In addition to applications in tissue engineering, to enhance their therapeutic efficacy, developing a cellular therapy using MSCs as attractive delivery vectors is the ultimate goal of this area of research. Genetic engineering methods to modify MSCs can be classified as those using viral transduction, nonviral transfection, or genome editing tools and techniques to overexpress therapeutic proteins that complement their innate properties (Figure 3) 43-46. A growing body of evidence indicates that the paracrine, homing, immunomodulatory, anti-inflammatory, and tissue repair properties of MSCs can be strengthened through genetic modification 47. As therapeutic agents and novel carriers, genetically modified MSCs target metastasis and efficiently provide a local high concentration of therapeutic agents that target a specific disease (Table 1). These strategies offer therapeutic dosages of MSCs and therapeutic agents at the target site, circumventing the problems with toxicities for repetitive systemic administration.

## Clinical Applications

### Methods

#### Search strategy

**Figure 4:**
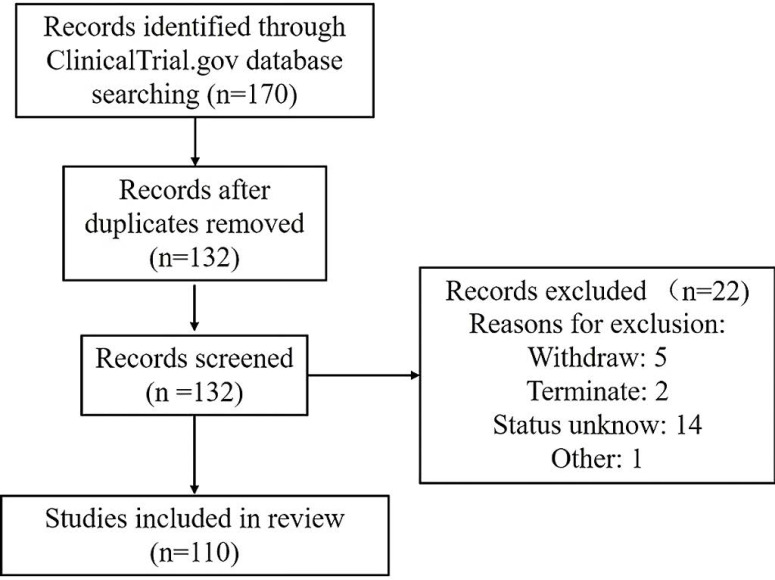
Framework for the selection of relevant clinical trials.

A comprehensive search of the ClinicalTrials.gov database from 2000 to January 19, 2021, was conducted according to Preferred Reporting Items of Systematic Reviews and Meta-analyses (PRISMA) guidelines. The keywords used to search for MSC-based therapy for lung disease in ClinicalTrials.gov were as follows: 1) Condition or disease: “acute respiratory distress” OR “acute respiratory syndrome” OR ARDS, “2019 novel coronavirus” OR “2019-nCoV” OR “COVID19” OR “interstitial pneumonia” OR “viral pneumonia” OR “virus pneumonia”, “bacterial pneumonia”, “chronic pulmonary diseases” OR “chronic obstructive pulmonary disease” OR COPD OR “emphysema”, “bronchopulmonary dysplasia” OR BPD, “idiopathic pulmonary fibrosis”, “pulmonary arterial hypertension”, “asthma”, “lung transplant reject”, “lung disease” and “pulmonary disease”; and 2) Other terms: “Mesenchymal stromal cells” OR MSC OR MSCs. This therapeutic review provides an evaluation of the use of MSCs in acute and chronic pulmonary disease treatment. A total of 170 clinical trials were initially found. After the exclusion of 38 duplicates and 22 trials of “unknown”, “terminated” and “withdrawn”, 110 trials focused on MSC therapy in pulmonary diseases were reviewed using Prisma Flow (Figure 4).

#### Aims and outcomes

This review included registered clinical trials that evaluated the safety and/or efficacy of MSCs administered to patients with lung diseases from any cause, either complete or ongoing. The use of MSCs as monotherapy and/or combined therapy was included. Additionally, one unregistered study with results was identified on PubMed and discussed briefly here. The primary outcomes were the comprehensive safety and efficacy evaluation of MSC use in pulmonary disease therapy. Secondary outcomes were changes in pulmonary function and biomarkers. All results collected from the studies were reported with the same measurements retrieved from the papers.

## Results

**Figure 5:**
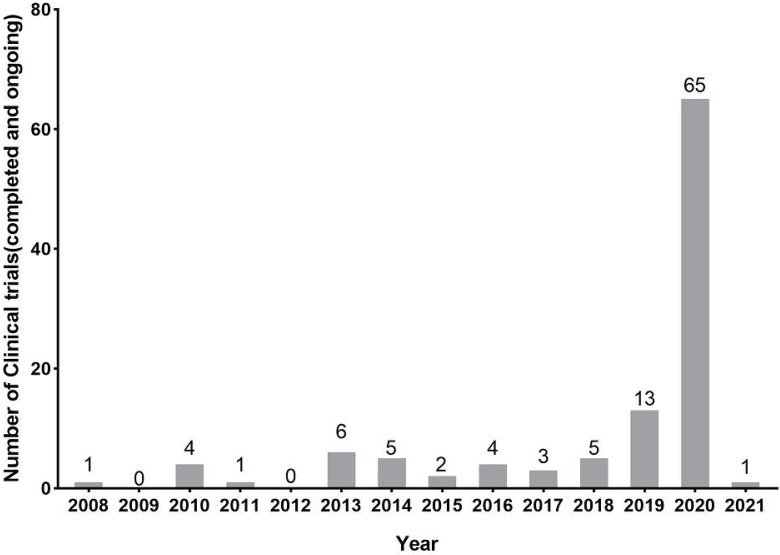
The number of registered clinical trials in MSCs for pulmonary diseases at Clinicaltrials.gov through chronological distribution from 2008 year. Data were obtained on January 2021.

**Figure 6:**
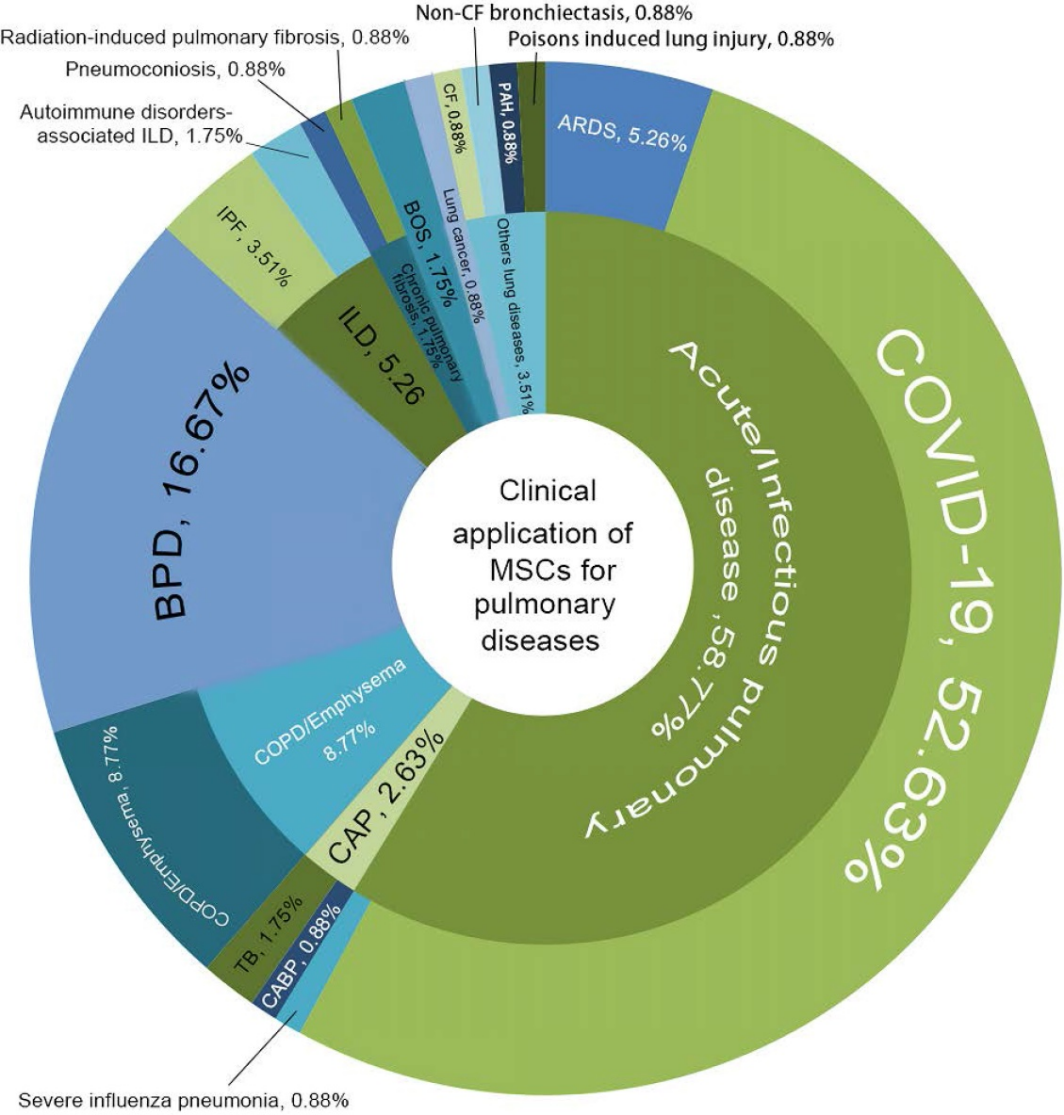
Clinical application of MSCs for pulmonary diseases. COVID-19, coronavirus disease 2019; ARDS, acute respiratory distress syndrome; CAP, community-acquired pneumonia; CABP, community-acquired bacterial pneumonia; TB, tuberculosis; COPD, chronic obstructive pulmonary disease; BPD, Bronchopulmonary dysplasia; ILD, interstitial lung diseases; IPF, idiopathic pulmonary fibrosis; BOS, bronchiolitis obliterans syndrome; PAH, pulmonary arterial hypertension; CF, cystic fibrosis.

**Figure 7:**
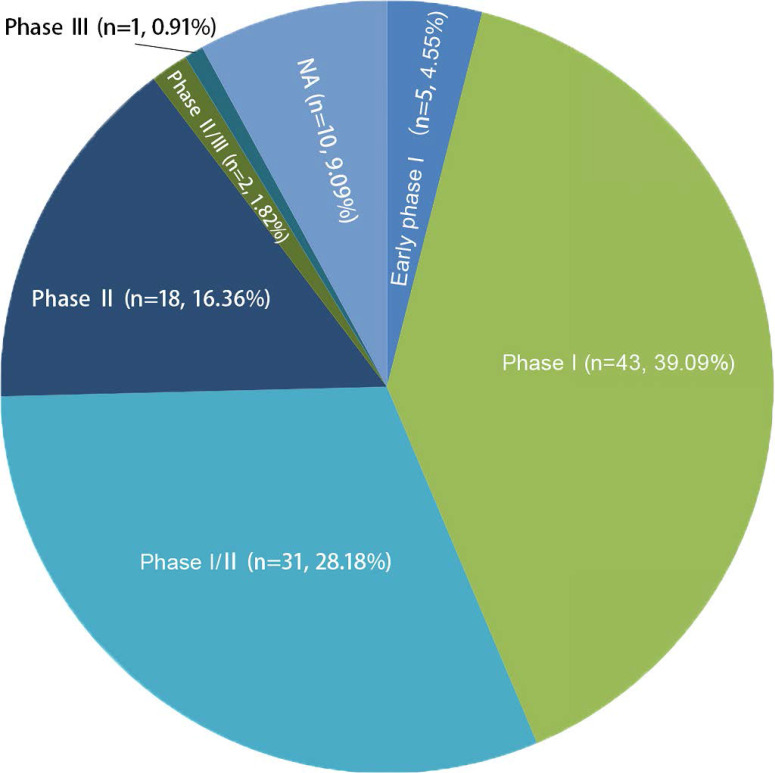
The phase numbers and percentage of registered clinical trials. Phase I and II clinical trials of MSCs for pulmnary diseases about 88% of the total number. NA, not applicable.

In general, new registrations of clinical trials with MSC-based therapy reached a peak in 2020, accompanied by one startling discovery of 58 registered MSC trials specifically targeting COVID-19 (Figure 5). The first clinical trial involving the use of MSCs for pulmonary disease was conducted in 2008, and the results were published in 2013 48. These clinical studies involved acute/viral pulmonary disease, CAP, COPD/emphysema, BPD, ILD, chronic pulmonary fibrosis, CLAD, BOS and lung cancer. Additionally, clinical trials are underway for cystic fibrosis (CF), non-CF bronchiectasis, pulmonary arterial hypertension (PAH), and even poison-induced lung injury (Figure 6). These clinical trials are listed in Table 2 (completed and published trials) and Table 3 (ongoing trials). The majority of clinical trials are still in Phase I (safety studies), Phase II (proof of concept for efficacy in human patients), or a mixture of Phase I/II, as shown in Figure 7.

### Acute/viral pulmonary disease

#### ARDS

ARDS is a devastating disorder characterized by acute and refractory hypoxia, noncardiogenic pulmonary edema, diffuse alveolar-capillary membrane damage, and reduced compliance 49. ARDS and pneumonia are interrelated in critically ill patients 49. Despite decades of research, there is still no effective pharmacotherapy for ARDS. Although some supportive care approaches have been established, ARDS remains devastating and life-threatening. ARDS constitutes a spectrum of increasingly severe acute respiratory failure with growing prevalence and high mortality and morbidity that increase with age 50,51.

To date, there have been 8 registered clinical trials using MSC- and MSC-derived exosomes for the treatment of ARDS (Table 2 and Table 3). In the first early-stage clinical trial, MSCs were utilized for the treatment of ARDS (NCT01902082) in Shaoxing Second Hospital of China between January and April 2013 52. The study population comprised 6 patients randomized to the MSC group and 6 patients randomized to the placebo group, in which the patients in the MSC group received a single intravenous dose of 1×10^6^ ADSCs per kilogram of weight. The results showed no infusion toxicities or serious adverse events related to MSC administration. However, the two groups were similar in the length of hospital stay, ventilator-free days, and ICU-free days within 28 days after the treatment. Subsequently, Wilson et al. 53 reported the START trial (NCT01775774), a Phase I, multicenter, open label, dose escalation pilot study designed to test the safety of a single-dose systemic injection of allogeneic BMSCs in patients with moderate to severe ARDS. Nine patients received intravenous infusions of BMSCs at a low dose (*n=3*, 1×10^6^ cells/kg), an intermediate dose (*n=3*, 5×10^6^ cells/kg) or a high dose (*n=3*, 10×10^6^ cells/kg). High dose BM-MSCs improved daily sequential organ failure assessment (SOFA) score compared to lower doses. However, no signifcant differences in inflammatory and endothelial injury markers were detected in any of the samples collected. The trial demonstrated that a single intravenous dose of MSCs of up to 1×10^6^ BMSCs/kg was well tolerated. Another Phase I trial (NCT02804945) have completed in June 2019. The participants received a maximum dose of 3×10^6^ cells per kilogram of weight intravenously. However, the result has not been posted yet. In addition, Chen et al. 54 reported that the transplantation of menstrual blood-derived MSCs could reduce mortality in patients with H7N9 virus-induced ARDS without adverse effects after a five-year follow-up period in China. Because H7N9 and COVID-19 share similar complications, MSC transplantation may be useful for treating COVID-19.

#### COVID-19/severe influenza

The cure of COVID-19 is essentially dependent on the patients' own immune system. When the immune system is over activated in an attempt to kill the virus, this can lead to the production of a large number of inflammatory factors, resulting in severe cytokine storm. The cytokine storm may induce organ damage followed by the edema, dysfunction of air exchange, ARDS, acute cardiac injury, and secondary infection, which may lead to death 55. Thus, preventing the severe acute respiratory infection and cytokine storm form of COVID-19 as the most dangerous phase of this disease can be helpful for the treatment and reduction of the death rate 56. In this regard, MSC-based immunomodulation treatment has been proposed as a suitable therapeutic approach, and several clinical trials have begun. More recently, a growing number of clinical investigations of cell-based therapies, primarily involving MSCs but also involving MSC-derived exosomes, have been initiated worldwide for COVID-19.

MSCs were utilized for the first time for the therapeutic application of COVID-19 pneumonia in Beijing YouAn Hospital, China, from Jan 23, 2020 to Feb 16, 2020 57. In this clinical study, seven confirmed COVID-19 patients received single dose of clinical grade MSCs (1×10^6^ cells per kilogram of weight). The pulmonary function and symptoms of these seven patients were significantly improved 2 days after MSC transplantation. Analysis of immune cells revealed that there was an increment of blood lymphocyte concentrations, Tregs and DCs with decreased NK cells. Meanwhile, the plasma level of C-reactive protein (CRP) and TNF-α was significantly decreased, while IL-10 and vascular endothelial-derived growth factor (VEGF), which correlated with pulmonary regeneration, increased in the MSC treatment group compared to the placebo control group. The satisfactory results of the MSCs therapy gave hope for more critically ill COVID-19 patient. Another clinical study is a case report of a 65-year-old woman diagnosed with critically ill-type COVID-19 along with acute respiratory failure and acute diarrhea on January 31, 2020 58. During the treatment, three doses of 5×10^7^/administration UCMSCs were used, 3 days apart. Stem cell therapy was used with conventional therapy to which the patient did not respond. After the third infusion, the patient was negative for SARS-CoV-2 and discharged with no side effects. Additionally, a case report study also described the therapeutic efficacy of the human umbilical cord Wharton's jelly-derived MSCs (hWJCs) (1×10^6^ cells per kilogram of weight) on a patient with COVID-19 pneumonia 59. This report suggested that the adoptive transfer therapy of hWJCs might be an ideal choice to be used for COVID-19 treatment.

While basic studies using MSC-derived exosomes have not been sufficiently performed for COVID-19, clinical studies using exosomes are in the planning stage or have recently been initiated. Recently, a pilot study using allogenic ADSC-derived exosomes for treating severe COVID-19 was completed in China (NCT04276987) 60. This trial is a Phase I, randomized, single-group assignment study whose primary objective is to explore the safety and efficiency of exosomes in the treatment of severe COVID-19 patients (Table 2). Moreover, there was a similar clinical trial had been registered in Russia. The COVID-19EXO trial (NCT04491240), a Phase I/II, randomized, open-label, parallel-group study, was completed. This trial enrolled 30 patients, and all eligible study subjects were randomized, double-blinded, to either one of the two treatment groups or placebo group. The patients in the treatment groups received inhalation of 3 ml of special solution containing 0.5-2×10^10^ exosomes twice a day for 10 days in combination with standard therapy. The primary outcome measure was the number of patients with nonserious and serious adverse events during the trial. Inspiringly, according to the results posted on ClinicalTrial.gov, no adverse events were registered 61.

Up to January 19, 2021, there were 58 registered clinical trials of MSC (*n=56*) and MSCs-dervied exosomes (*n=2*), of which 33 are active and recruiting patients and six have completed their trials (Table 2 and Table 3). The sources of MSCs are umbilical cord (*n*=21), Wharton's jelly (*n*=3), placental tissue (*n*=1), bone marrow (*n*=9), adipose tissue (*n*=6), dental pulp (*n*=1), olfactory mucosa (*n*=1), and unmentioned origin (*n*=14) (Table 2 and Table 3). The first trial was registered on Feb 5, 2020 by Beijing 302 Hospital. This phase І clinical trial (NCT04252118) was done to inspect the safety of UCMSCs therapy for pneumonia patients infected with SARS-CoV-2 62. The second trial (NCT04269525) was registered on Feb 13, 2020 by Zhongnan Hospital. This phase Ⅱ trial was being conducted to assess the role of UCMSCs (100×10^6^ cells/time at D1, D3, D5, D7) in treating COVID-19 pneumonia 63.

The present preliminary clinical data reveal that MSCs succeed in managing severe and critically severe COVID-19 patient, and have a benefit in reducing inflammation, improving pulmonary function, and reducing death in COVID-19 patients. The factors considered to be vital for effective treatment include the route, timing, dose, volume, source, and duration of the MSC administration. Adequately powered clinical trials are urgently needed to test clinical outcomes in patients with COVID-19.

### CAP

#### Community-acquired bacterial pneumonia

Community-acquired bacterial pneumonia (CABP), as an acute lung infection, can lead to sepsis and is associated with high mortality rates in patients presenting with shock and/or respiratory failure who require mechanical ventilation and admission to intensive care units, thus reflecting the limited effectiveness of current therapy 64,65. Very recently, Laterre et al. 66 first reported an ongoing Phase I/II, randomized, double-blind, multicenter trial (NCT03158727) to assess the safety and efficacy of expanded allogeneic ADSCs for the treatment of patients with severe CABP (sCABP) admitted to the ICU. The study was initiated in January 2017 and is expected to be completed by December 2021 (Table 3).

#### Tuberculosis

Tuberculosis (TB) remains an important cause of CAP. Mycobacterium tuberculosis has developed the ability to continually resist antitubercular agents. Multidrug-resistant TB (MDR-TB), defined by resistance to isoniazid and rifampicin, the two front-line antimicrobial drugs used to treat TB, presents one of the most urgent and difficult challenges facing global TB control 67. The first open-label Phase I clinical trial of 30 MDR-TB and extensively drug-resistant TB patients who received single-dose autologous bone marrow-derived MSCs (1×10^6^ cells per kilogram of weight) was conducted in 2010 by a specialist center in Minsk, Belarus, and the results were published in 2014 68. There were no serious adverse events reported. Subsequently, in a small cohort study comprising 36 patients with MDR TB, intravenous infusions of autologous BM-MSCs were administered 4 weeks after starting TB treatment 69. The results showed that autologous transplantation of MSCs could vastly improve outcomes for 81% of MDR-TB patients. This result could revolutionize therapy options and have strong implications for future directions of MDR-TB therapy research.

### COPD/Emphysema

COPD is an umbrella term used to describe chronic lung diseases, such as emphysema and chronic bronchitis, which cause limitations in airflow 70. The disease burden from COPD, in contrast to that of TB, appears to be growing, despite the development of new therapeutics such as long-acting antimuscarinic agents, long-acting β-agonists, inhaled corticosteroids, and phosphodiesterase inhibitors 71. Interest in using MSCs for the treatment of COPD or emphysema has translated into clinical trials. The first Phase II clinical trial (NCT00683722) involved the use of allogeneic BMSCs for the treatment of moderate-to-severe COPD from May 20, 2008, to August 24, 2010 48. Thirty patients received four monthly infusions (100×10^6^ cells/infusion) and completed the 2-year follow-up. This trial demonstrated that systemic administration of multiple doses of MSCs appears to be safe and may decrease inflammation in an older, comorbid population of patients with compromised lung function due to moderate to severe COPD.

In addition, Stolk et al. 72 reported another Phase I clinical trial (NCT01306513) that aimed to study the safety and feasibility of intravenous administration of autologous BMSCs to patients with severe emphysema. Seven patients received bone marrow aspiration for BMSC collection, while the first underwent lung volume reduction surgery (LVRS) on one lung. The second LVRS on the contralateral lung was preceded by two intravenous infusions of autologous BMSCs (1-2×10^6^ cells/kg). After LVRS and MSC infusions, alveolar septa showed a 3-fold increased expression of the endothelial marker CD31. One year after the second LVRS, all patients presented increased forced expiratory volume in 1 second (FEV_1_) and body weight and changes in lung densitometry compared to their own values before the first LVRS. The results showed that autologous MSC administration in patients with severe emphysema is feasible and safe. However, a main limitation of the study was the lack of a placebo group. At present, there are 10 registered clinical trials using MSCs for the treatment of COPD or emphysema (Table 2 and Table 3). Moreover, with respect to cellular sources, only controlled trials with a strict comparison between different tissues might determine the suitability and efficacy of specific cell types to treat COPD or emphysema.

### BPD

BPD is the most prevalent respiratory disorder among infants born extremely preterm and is characterized by the arrest of alveolarization, fibroblast activation, and inflammation 73. It is one of the leading causes of chronic lung disease in children 74. The pathogenesis of BPD involves multiple prenatal and postnatal mechanisms affecting the development of very immature lungs. Their combined effects alter the lung's morphogenesis, disrupt capillary gas exchange in the alveoli, and lead to the pathological and clinical features of BPD 75.

Chang et al. 76 reported the first Phase I dose-escalation clinical trial (NCT01297205) in 2014 to evaluate the safety and efficacy of intratracheal transplantation of human UCMSCs in preterm infants at high risk for BPD. This trial demonstrated that the treatment was well tolerated, without serious adverse effects or dose-limiting toxicity: all 9 infants who underwent MSC transplantation survived, and only 3 of these infants developed moderate BPD. A two-year follow-up (NCT01632475) by the same researchers indicated that one of 9 infants in the MSC group died of *Enterobacter cloacae* sepsis at 6 months, and 8 infants survived without any transplantation-related adverse outcomes 77. Intratracheal transplantation of allogeneic UCMSCs in preterm infants is safe and feasible. The next Phase II clinical trial (NCT03392467) and follow-up (NCT04003857) for intratracheal instillation of UCMSCs to preterm infants with BPD are ongoing 78,79. Recently, Wu et al. 80 reported the first randomized, single-center, open-label, dose-escalation, Phase II trial (NCT03601416) using MSCs intravenously administered in children with severe BPD. In this study, the safety and efficacy of treatment with low- (*n*=24, 2.5×10^6^ cells/kg) and high-dose (*n*=24, 5×10^6^ cells/kg) intravenous infusions of allogeneic UCMSCs were compared with those of traditional supportive treatments for BPD. These results will provide new evidence of MSC-based therapy for severe BPD.

### ILD

#### IPF

IPF is the most lethal ILD, characterized by fibrosis following failed epithelial repair and chronic progressive scarring of the lungs 81. Although the precise etiology is unknown, a number of risk factors may contribute to disease development, including smoking, drug exposure, infectious agents, and genetic predisposition 82. Currently, its associated mortality remains high, and no effective pharmacotherapy or artificial ventilation and transplantation exists. The administration of MSCs is investigated as a new therapeutic method for IPF 83.

The first pilot IPF clinical trial (NCT01385644) with placenta-derived MSC therapy was conducted in 2010 in Australia, and the results were published in 2014 84. In this single-center, nonrandomized, dose escalation Phase Ib study, four out of the 8 patients participating in the trial received intravenous infusion of placenta-derived MSCs at 1×10^6^ cells/kg, and another 4 patients received 2×10^6^ cells/kg by the same delivery. Both dose schedules were well tolerated, with only minor and transient acute adverse effects. At 6 months postinfusion, most adverse events of this trial were mild and selflimiting, and lung function and computed tomography (CT) fibrosis scores were all unchanged from baseline, with no evidence of worsening fibrosis 84. These results demonstrated that intravenous MSCs for patients with moderately severe IPF are feasible and have a good short-term safety profile. Subsequently, in a Phase I/II clinical trial (NCT02594839), twenty patients with a rapid progressive course of severe to moderate IPF were randomized into two groups: one group received two intravenous doses of allogeneic BMSCs (2×10^8^ cells) every 3 months (total amount: 1.6×10^9^ cells). After the study was completed, no significant adverse effects were found in the MSC-administrated group, and they were observed having a better outcome for the 6-min walk test distance, for DLCO in 26 weeks, and for forced ventilation capacity in 39 weeks compared with the placebo group 85. Therapy with high doses of BMSCs is a promising method for reducing rapid pulmonary function decline in patients with IPF. Another trial (NCT02013700) also supports the safety of a single infusion of BMSCs in patients with mild-moderate IPF 86. Moreover, the authors nicely discuss the limitations of the study, which include the small sample size (nine patients), the lack of randomization, and the absence of a placebo control arm for comparison. These trials demonstrate that therapy with high doses of allogeneic MSCs is a safe and promising method for reducing disease progression in patients with IPF. Ultimately, we need a large number of Phase II/III clinical trials of MSCs for IPF to evaluate their efficacy.

#### ILD associated with autoimmune disorders

ILD can manifest as a pulmonary complication of an underlying autoimmune and connective tissue disease (CTD-ILD), such as systemic sclerosis (SSc-ILD). ILD associated with SSc, together with pulmonary hypertension, represents the most common cause of death 87. The most common agents currently utilized for the treatment of CTD-ILD include corticosteroids, azathioprine (AZA), mycophenolate mofetil (MMF) and cyclophosphamide (CYC) 88,89. In recent years, researchers have attempted to determine more about the safety of MSC treatment or CTD-ILD, especially as MSCs can counteract the three main pathogenic axes of the disease: fibrosis, angiogenic defects, and autoimmunity 90. The first Phase I trial (NCT03929120) designed to evaluate the safety of MSCs for patients with CTD-ILD is ongoing 91. Another clinical trial is ongoing (NCT04432545) in Colombia, which aims to evaluate the therapeutic effects of allogeneic MSC infusion as a treatment in patients with SSc-ILD refractory to conventional therapy 92 (Table 3).

### Chronic pulmonary fibrosis

#### Pneumoconiosis

Pneumoconiosis is a kind of lung disease caused by inhalation of dust, such as silica (commonly named siliconosis), coal and rock dust and is characterized by inflammation, coughing, and fibrosis 93. Early pneumoconiosis may be asymptomatic, but advanced stages of pneumoconiosis result in airflow limitation, hypoxia, pulmonary hypertension, respiratory or heart failure, and premature death, even without further exposure to the dust 94. Currently, there is no effective drug treatment. The first Phase I clinical trial (NCT02668068) using UCMSCs for pneumoconiosis was registered in January 2016 95. This study was completed in China and observed and evaluated the safety and efficacy of combined large volume WLL with MSC transplantation for the treatment of pneumoconiosis. However, no results have been reported yet.

#### Radiation-induced pulmonary fibrosis

The lung is a radiosensitive organ, and pulmonary damage after high-dose radiation can cause radiation pneumonitis in the early stages and pulmonary fibrosis later on 96. Effective treatments for improving patient prognosis are lacking. A Phase I, open, single-center, nonrandomized clinical study (NCT02277145) on radiation-induced pulmonary fibrosis treated with umbilical cord-derived MSCs was completed in December 2018 97. Patients received 1×10^6^ cells per kilogram of weight of clinical grade UCMSCs injected via fiberoptic bronchoscopy after full lavage of the localized lesions. However, no results of this trial have been reported.

### BOS

BOS, characterized by persistent airflow obstruction, is a devastating complication after lung transplantation 98 and allogeneic hematopoietic stem cell transplantation (allo-HSCT) 99. The key clinical feature of BOS is the development of airway obstruction with a reduction in FEV_1_ that does not respond to bronchodilators. The first clinical trial (NCT01175655) for patients with BOS after lung transplantation treated with allogeneic MSCs was published in July 2017 100. In this trial, a total of ten lung transplant recipients diagnosed with BOS received MSC infusions at a dose of 2×10^6^ cells per kilogram of weight for each infusion twice weekly for 2 weeks. Study data confirmed the feasibility and safety of such intravenous delivery of allogeneic MSCs in patients with advanced BOS. Another multicenter, open-label, Phase I/II, prospective cohort study (NCT02543073) evaluated the safety and efficacy of allogeneic BMSCs for allo-HSCT associated BOS recipients 101. In the MSC group, MSCs were intravenously given at a median dose of 1×10^6^ cells per kilogram of weight once weekly for 4 consecutive weeks as a cycle. If tolerated, a second cycle was given at a 2-week interval. The outcome of the study revealed that MSCs may be a safe and effective therapy for BOS patients after allo-HSCT.

### Lung cancer

As genetically modified vectors, combining the tumor-homing capacity of MSCs and genetic engineering of the cells to express tumor necrosis factor (TNF)-related apoptosis inducing ligand (TRAIL) will enable the specific targeting of cancer stem cells (CSCs), which would be an attractive cytotherapeutic option for cancer 102. A Phase I clinical trial (NCT03298763) of MSC-TRAIL for lung cancer is ongoing in the UK, which aims to establish the recommended MSC-TRAIL dose when given in combination with cisplatin/pemetrexed chemotherapy in metastatic non-small cell lung cancer (NSCLC) patients 103. The study was initiated in March 2019 and is expected to be completed by September 2025.

### Other lung diseases

#### CF

CF is a common autosomal recessive disease that primarily affects the lungs and digestive system and is characterized by obstruction of airways, microbial infection, digestive disorders, and other complications due to mutations in CF transmembrane conductance regulator (CFTR) 104. MSCs could be used to restore abnormal CFTR function. Moreover, the ability of MSCs to secrete the antimicrobial peptide LL-37, which is associated with the capacity to slow bacterial growth 105, will be a promising treatment for MSCs in patients with CF. The CEASE-CF trial (NCT02866721), a Phase І, single-center, open label, dose escalation study, was completed in April 2020, and the results have not yet been reported 106.

#### Non-CF bronchiectasis

Non-CF bronchiectasis is a syndrome of chronic inflammation leading to dilatation of airways and structural lung damage, which imposes a significant burden on patients. The observed cause of death is due primarily to bronchiectasis or related respiratory failure 107. To demonstrate the safety of BMSCs in patients with non-CF bronchiectasis receiving standard of care therapy and to explore treatment efficacy, a Phase I investigation (NCT02625246) was completed in May 2019; however, the results are not available 108.

#### PAH

PAH is a rare, progressive disorder characterized by increased blood pressure in the arteries of the lungs. Although PAH is manageable, there is no effective therapy able to reduce mortality 109. One trial (NCT04055415) evaluating the safety and initial impact of a single intravenous dose of a cell-based product made from allogeneic ADSCs (1×10^6^ cells per kilogram of weight) to treat PAH is ongoing 110.

#### Poison-induced lung injury

Sulfur mustard (SM) is a potent alkylating toxic chemical compound that targets several organs, especially the lungs. Acute lung injury due to SM inhalation causes the formation of airway fibrin casts that obstruct airways at multiple levels, which is associated with chronic obstructive pulmonary deficiency, leading to acute respiratory failure and death 111. Currently, effective medical countermeasures for SM are lacking. Ghazanfari et al. 112 showed that short-term SM exposure led to a decline in circulating MSC count after more than two decades. The lower number of peripheral MSCs in SM-exposed patients was not affected by taking corticosteroids or antibiotics, but comorbidities are probably involved in MSC frequency. In 2017, Nejad-Moghaddam et al. 113 reported a clinical trial (NCT02749448) using multiple doses of ADSC therapy for a male patient with SM-exposed lung injury at the Chemical Injuries Research Center, Baqiyatallah University of Medical Sciences, Tehran, Iran. The patient received 100×10^6^ cells every 20 days for a total of 4 injections within a 2-month period, and precise evaluations were performed. The results indicated that systemic ADSC administration appears to be safe and shows promising results with improvement of the patient's physical activity and 6MWT, FEV_1_ and COPD assessment test (CAT) scores.

## Discussion

Accumulating evidence supports MSC-based therapy as a promising therapeutic strategy in clinical trials of refractory and unmanageable pulmonary illnesses for targeting viral infection, fibrotic processes, and excessive inflammatory response, as well as combating organ failure 114. Systemically infused MSCs have been found to migrate directly to the lungs, where they can ameliorate cytokine release syndrome, protect alveolar epithelial cells, repair injured airways, aid in alveolar fluid clearance, promote epithelial and endothelial recovery, resist pulmonary fibrosis, reduce the risk of allograft rejection, and improve lung function by secreting many kinds of factors and modulating multiple biological processes of the immune response, which are great benefits for treating severe pulmonary disease 115-117.

To date, several clinical trials have evaluated the safety, tolerance, and severe adverse events of MSC administration, and many clinical trials are still ongoing. Published phase I/II clinical trials seem to reasonably prove the safety and clinical improvement of MSC administration, with no significant adverse events, in acute and chronic lung diseases. Given that most clinical trials are in the early phase, undoubtedly, placebo-controlled, multicenter, more randomized large-scale phase II/III trials are needed to reach more convincing conclusions regarding the safety, effect sustainability and adverse effects of MSC therapies 118. Additionally, evaluations of long-term safety or efficacy and the duration of local or systemic MSC transplantation are required.

Currently, the optimum therapeutic dosage of MSCs for treating lung diseases is unknown. In these published clinical trials, a wide dosage array of 1×10^6^ cells~10×10^6^ cells per kg of weight was used. Wilson et al. 53 reported that the application of three doses was administered in three cohorts (1×10^6^ cells/kg, 5×10^6^ cells/kg, and 10×10^6^ cells/kg) in patients with ARDS, resulting in a corresponding reduction in the lung injury score of 30%, 36%, and 45%, respectively, and that the maximal dosage was well tolerated by patients. Recent studies have shown that MSC-based therapy significantly dampens cytokine storms in critically ill COVID-19 patients by negatively regulating the immune response. Accordingly, an optimal dosage of transplanted MSCs should be clearly defined, with the aim of finding the right balance between their beneficial and undesired effects, which could occur due to excessive immunosuppression 119. In addition, the therapeutic effects of MSC administration should be carefully monitored since the differentiation potential, capacity for migration, immunomodulation and maximal life span of transplanted MSCs decline with increasing age.

## Conclusion

MSC-based therapy approaches for lung diseases and critical illness continue to evolve at a rapid pace and offer hope for treating these devastating and currently incurable diseases. Further studies are expected to improve the standardization of MSC treatment protocols in terms of the donor source (autologous vs. allogeneic), sources of MSCs, MSCs culture status (fresh vs. cryopreserved/thawed), manufacturing protocols, quality control provisions, routes of delivery (systemic vs. local), and cell dosing. Additionally, strict patient inclusion/exclusion criteria should be defined, well-designed and controlled clinical trials should be performed, and rigorous ethical considerations must ensure patient safety before MSCs can be used in large-scale and long-term clinical applications for cell therapy.

## Figures and Tables

**Table 1:** Genetically modified mesenchymal stem cells

Type of Genes	Molecules
Costimulatory molecules	*CTLA-4Ig, ICOSIg, OX40Ig, PD-1*
Chemokines	*CXCR2, CXCR3, CXCR4,*…
Enzyme	*hTERT, ILK, TIMP2,* …
Growth factor	*BDNF, FGF, HGF, VEGF,* …
IFN	*IFN-β, IFN-γ*
Interleukin	*IL-2, IL-4, IL-10, IL-17, IL-33, IL-35,* …
Tumor necrosis factor	*TNFR, TRAIL,* …
Transcription factor	*HIF-1α, SOX,*…
Transforming growth factor	*BMP, HO-1, TGF-β3,* …
RNA	*miR-9-5p, miR-10a, miR-215b, miR-486,* …
Other proteins	*ApoJ, PEDF, TLR4, TSP-4,*…

ApoJ, apolipoprotein J; BMP, bone morphogenetic protein; BDNF, brain-derived neurotrophic factor; CTLA-4, cytotoxic T lymphocyte-associated antigen-4; CXCR, C-X-C receptor; FGF, fibroblast growth factor; HGF, hepatocyte growth factor; HIF-1α, hypoxia inducible factor 1α; HO-1, heme oxygenase 1; hTERT, human telomerase reverse transcriptase; ICOS, inducible costimulatory; IFN, interferon; ILK, integrin-linked kinase; PD-1, programmed death-1; PEDF, pigment epithelial-derived factor; SOX, sex-determining region Y-type high-mobility-group-box; TIMP2, recombinant tissue Inhibitors of metalloproteinase 2; TLR4, Toll-like receptor 4; TRAIL, tumor necrosis factor-related apoptosis inducing ligand; TSP-4, thrombospondin 4; VEGF, vascular endothelial growth factor.

**Table 2:** Completed or published clinical trials of MSCs for pulmonary disease by January 2021

No.	Condition or disease	Clinical trial No.	Study								
			Phase	MSCs source	Title	Enrollment	Delivery and Dose	Results	Start Date	Completion Date	Locations
1	COVID-19	NCT04573270	I	UCMSCs	Mesenchymal Stem Cells for the Treatment of COVID-19	40	IV	No results posted	April 2020	September 2020	United States
2	COVID-19	NCT04288102	II	UCMSCs	Treatment With Human Umbilical Cord-derived Mesenchymal Stem Cells for Severe Corona Virus Disease 2019 (COVID-19)	100	IV, 3 does of MSCs (400×10^6^ cells/time at D0, D3, D6)	Safty, ↑6-MWT; improvement in whole lung lesion volume from baseline to day 28	March 2020	July 2020	China
3	COVID-19	NCT04355728	I-II	UCMSCs	Use of UC-MSCs for COVID-19 Patients	24	IV, 2 doses of 100×10^6^ cells/time	No results posted	April 2020	October 2020	United States
4	COVID-19	NCT04492501	NA	BMSCs	Investigational Treatments for COVID-19 in Tertiary Care Hospital of Pakistan	600	IV, single dose of 2×10^6^ cells/kg BW	No results posted	April 2020	July 2020	Pakistan
5	COVID-19	NCT04276987	I	MSCs-derived exosomes	A Pilot Clinical Study on Inhalation of Mesenchymal Stem Cells Exosomes Treating Severe Novel Coronavirus Pneumonia	24	Inhalation, 5 times of 2×10^8^ nano vesicles/3 ml at D1, D2, D3, D4, D5	No results posted	February 2020	July 2020	China
6	COVID-19	NCT04491240	I-II	MSCs-derived exosomes	Evaluation of Safety and Efficiency of Method of Exosome Inhalation in SARS-CoV-2 Associated Pneumonia.	30	Inhalation, Twice a day during 10 days inhalation of 3 ml 0.5-2×10^10^ nanoparticles	Safty	July 2020	October 2020	Russian Federation
7	ARDS	NCT01775774	I	Allogeneic BMSCs	Human Mesenchymal Stem Cells For Acute Respiratory Distress Syndrome	9	IV, dose-escalation with 3 cohorts with 3 subjects/cohort who receive doses of 1, 5 and 10×10^6^ cells/kg BW	Safty	July 2013	February 2015	United States
8	ARDS	NCT02804945	I	NA	Mesenchymal Stem Cells (MSCs) for Treatment of Acute Respiratory Distress Syndrome (ARD) in Patients With Malignancies	20	IV, 3×10^6^ cells/kg BW	No results posted	February 2017	June 2019	United States
9	COPD	NCT00683722	II	NA	PROCHYMAL™(Human Adult Stem Cells) for the Treatment of Moderate to Severe Chronic Obstructive Pulmonary Disease (COPD)	62	IV, 100×10^6^ cells on days 0, 30, 60, and 90	↓ Circulating CRP levels at 1 month after the first infusion; No statistically significant differences in FEV_1_	May 2008	August 2010	US
10	COPD	NCT01306513	I	Autologous BMSCs	Safety and Feasibility Study of Administration of Mesenchymal Stemcells for Treatment of Emphysema	10	IV, twice infusion (1-2×106 cells/kg), one week apart	Safty, ↑3-fold increased expression of the endothelial marker CD31	October 2010	November 2012	NA
11	COPD	NCT02216630	I-II	Autologous ADSCs	Safety and Efficacy of Adipose Derived Stem Cells for Chronic Obstructive Pulmonary Disease	26	IV, ADSCs are isolated from 100 cc of patients liposuction fat	No results posted	August 2014	July 2017	US
12	COPD	NCT01953523	NA	Autologous BMSCs	Safety and Clinical Outcomes Study: SVF Deployment for Orthopedic, Neurologic, Urologic, and Cardio-pulmonary Conditions.	3000	IV	No results posted	September 2013	January 2017	US
13	BPD	NCT01297205	I	UCMSCs	Safety and Efficacy Evaluation of PNEUMOSTEM® Treatment in Premature Infants With Bronchopulmonary Dysplasia	9	Intratracheal, low dose: 1×107 cells/kg BW; high dose: 2×107 cells/kg BW	Intratracheal transplantation of up to 2×10^7^ cells/kg of hUCB-derived MSCs in preterm infants may be safe and feasible	December 2010	December 2011	Korea
14	BPD	NCT01632475	NA	NA	Follow-Up Study of Safety and Efficacy of Pneumostem® in Premature Infants With Bronchopulmonary Dysplasia (NCT01297205)	9	NA	No infant was rehospitalized because of respiratory infection after 12 months; No infant showed any abnormality, such as a visible mass lesion, in the chest radiograph taken at visit 3	September 2011	September 2026	Korea
15	BPD	NCT02023788	NA	NA	Long-term Safety and Efficacy Follow-up Study of PNEUMOSTEM® in Patients Who Completed PNEUMOSTEM® Phase-I Study	8	NA	No results posted	April 2014	October 2016	Korea
16	BPD	NCT02381366	I-II	UCMSCs	Safety and Efficacy of PNEUMOSTEM® in Premature Infants at High Risk for Bronchopulmonary Dysplasia (BPD) - a US Study	12	Intratracheal, low dose group (3 patients: 1.0×10^7^cells/kg BW); high dose group (6 patients: 2 ×10^7^ cells/kg BW)	No evidence of lung pathology was found on serial chest radiographs, other than typical changes associated with BPD	March 2015	May 2018	US
17	BPD	NCT01828957	II	UCMSCs	Efficacy and Safety Evaluation of Pneumostem® Versus a Control Group for Treatment of BPD in Premature Infants	69	Intratracheal, single dose of MSCs (1.0×10^7^ cells/kg BW)	No results posted	April 2013	August 2015	Korea
18	BPD	NCT01897987	NA	NA	Follow-up Safety and Efficacy Evaluation on Subjects Who Completed PNEUMOSTEM® Phase-II Clinical Trial (NCT01828957)	62	NA	No results posted	January 2014	March 2020	Korea
19	IPF	NCT01385644	I	Placental-MSCs	A Study to Evaluate the Potential Role of Mesenchymal Stem Cells in the Treatment of Idiopathic Pulmonary Fibrosis	8	IV, 1×10^6^ cells/kg BW(4 patients); 2×10^6^ cells/kg BW(4 patients)	FVC, DLCO, 6MWD and CT fibrosis score were unchanged compared with baseline at 6 months; no evidence of worsening fibrosis	October 2010	May 2013	Australia
20	IPF	NCT02013700	I	AllogeneicBMSCs	Allogeneic Human Cells (hMSC)in Patients With Idiopathic Pulmonary Fibrosis Via Intravenous Delivery (AETHER)	9	IV, a single does of 200×10^6^ cells	↓ 3.0% in FVC and ↓ 5.4% in diffusing capacity of the lungs for carbon monoxide by 60 weeks postinfusion; no serious adverse effects	November 2013	November 2016	US
21	IPF	NCT02594839	I-II	AllogeneicBMSCs	Safety and Efficacy of Allogeneic Mesenchymal Stem Cells in Patients With Rapidly Progressive Interstitial Lung Disease	20	IV, twice of 2×10^8^ cells every 3 months, for one year; a total amount of 1.6×10^9^ MSCs	↑6MWD in 13 weeks; ↑ DLCO in 26 weeks;↑7.8% from baseline FVC; no significant adverse effects	February 2013	January 2018	Russian Federation
22	IPF	NCT01919827	I	AutologousBMSCs	Study of Autologous Mesenchymal Stem Cells to Treat Idiopathic Pulmonary Fibrosis	17	Endobronchial infusion	No results posted	March 2013	May 2018	Spain
23	BOS	NCT02543073	I	NA	MSC for Treatment of Interstitial Lung Disease After Allo-HSCT	81	IV, 1×10^6^ cells/kg once weekly for 4 weeks	No serious adverse events. Better change in FEV_1_ rate of decline; ↑IL-10-producing CD5+B cells	September 2014	June 2018	China
24	BOS	NCT01175655	I	NA	A Study to Evaluate the Potential of Mesenchymal Stromal Cells to Treat Obliterative Bronchiolitis After Lung Transplantation (MSC in OB)	10	IV, 2×10^6^ cells/kg BW, twice weekly for 2 weeks	Safety	February 2010	July 2016	Australia
25	CF	NCT02866721	Ӏ	NA	Safety and Tolerability Study of Allogeneic Mesenchymal Stem Cell Infusion in Adults With Cystic Fibrosis (CEASE-CF)	14	IV, single dose, one time infusion of one of the following doses:1×10^6^, 3×10^6^, 5×10^6^ cells/kg BW.	No results posted	August 2016	August 2020	United States
26	Pneumoconiosis	NCT02668068	I	UCMSCs	A Study on Pneumoconiosis Treated With Whole-lung Lavage Combined With Mesenchymal Stem Cells	80	IV, 1×10^6^cells/kg BW	No results posted	January 2016	March 2019	China
27	Radiation-induced pulmonary fibrosis	NCT02277145	I	UCMSCs	A Study on Radiation-induced Pulmonary Fibrosis Treated With Clinical Grade Umbilical Cord Mesenchymal Stem Cells	10	IV, 1×10^6^cells/kg BW	No results posted	October 2014	December 2018	China
28	Non-CF bronchiectasis	NCT02625246	I	BMSCs	Safety and Potential Efficacy of Human Mesenchymal Stem Cells in Non-Cystic Fibrosis Bronchiectasis (CELEB)	6	IV, group 1: 3 patients, 20×10^6^cells; group 2: 3 patients, 100×10^6^cells;	No results posted	February 2016	May 2019	United States
29	Poisons induced lung injury	NCT02749448	I	ADSCs	Mesenchymal Stem Cells Therapy for Treatment of Airway Remodeling in Mustard Patients	10	IV, 100×10^6^ cells every 20 days for a total of 4 injections	Safty, ↑ 6MWD, FEV_1_ and COPD assessment test scores	February 2015	February 2017	NA

NA, not applicable; IV, intravenously; BW, body weight; COPD, chronic obstructive pulmonary disease; BPD, bronchopulmonary dysplasia; IPF, idiopathic pulmonary fibrosis; BOS, bronchiolitis obliterans syndrome; CF, cystic fibrosis; MSCs, mesenchymal stromal cell; BMSCs, bone marrow-derived MSCs; UCMSCs, umbilical cord-derived MSCs; ADSCs, adipose tissue-derived MSCs; CRP, C-reactive protein; FEV_1_, forced expiratory volume-one second; FVC, forced vital capacity; 6MWD, 6-min walk distance; CT, computed tomography; DLCO, diffusing capacity for carbon monoxide.

**Table 3:** 81 ongoing clinical trials investigated for the MSCs treatment of pulmonary diseases

NO.	Condition or disease	Clinical trials No.	Study								
			Statue	Phase	MSCs source	Title	Enrollment	Intervention/treatment	Start Date	Completion Date	Country
1	COVID-19	NCT04366063	Recruiting	II-III	NA	Mesenchymal Stem Cell Therapy for SARS-CoV-2-related Acute Respiratory Distress Syndrome	60	IV, 100×10^6^ cells/kg BW	April 2020	December 2020	Iran
2	COVID-19	NCT04371393	Active, not recruiting	III	NA	MSCs in COVID-19 ARDS	223	IV, 2×10^6^ cells/kg BW	April 2020	February 2022	United States
3	COVID-19	NCT04361942	Recruiting	II	NA	Treatment of Severe COVID-19 Pneumonia With Allogeneic Mesenchymal Stromal Cells (COVID_MSV)	24	IV, 1×10^6^ cells/kg BW	May 2020	December 2020	Spain
4	COVID-19	NCT04252118	Recruiting	I	NA	Mesenchymal Stem Cell Treatment for Pneumonia Patients Infected With COVID-19	20	IV, 3.0×10^6^ MSCs	January 2020	December 2021	China
5	COVID-19	NCT04315987	Not yet recruiting	II	NA	NestaCell® Mesenchymal Stem Cell to Treat Patients With Severe COVID-19 Pneumonia	90	IV, 20×10^6^ cells/kg WB	June 2020	August 2020	Brazil
6	COVID-19	NCT04525378	Recruiting	I	NA	MSC-based Therapy in COVID-19-associated Acute Respiratory Distress Syndrome	20	IV, low dose(25×10^6^); intermediate dose (50×10^6^); high dose (100×10^6^)	July 2020	October 2020	Brazil
7	COVID-19	NCT04629105	Recruiting	I	NA	Regenerative Medicine for COVID-19 and Flu-Elicited ARDS Using Longeveron Mesenchymal Stem Cells (LMSCs) (RECOVER)	70	IV, 3 doses of 100×10^6^ MSCs	July 2020	July 2025	United States
8	COVID-19	NCT04467047	Not yet recruiting	I	NA	Safety and Feasibility of Allogenic MSC in the Treatment of COVID-19	10	IV, 1×10^6^ MSCs/kg BW	July 2020	December 2020	NA
9	COVID-19	NCT04466098	Recruiting	II	NA	Multiple Dosing of Mesenchymal Stromal Cells in Patients With ARDS (COVID-19)	30	IV, 300×10^6^ MSC	July 2020	December 2021	United States
10	COVID-19	NCT04537351	Recruiting	I-II	NA	The MEseNchymal coviD-19 Trial: a Pilot Study to Investigate Early Efficacy of MSCs in Adults With COVID-19	24	IV, 2×10^6^ cells/kg BW (maximum of 200 million)	August 2020	March 2021	Australia
11	COVID-19	NCT04615429	Recruiting	II	NA	Clinical Trial to Assess the Efficacy of MSC in Patients With ARDS Due to COVID-19	20	1x10^6^ cells/kg BW	Septembe 2020	January 2022	Spain
12	COVID-19	NCT04524962	Recruiting	I-II	NA	Study of Descartes-30 in Acute Respiratory Distress Syndrome	30	NA	September 2020	September 2022	United States
13	COVID-19	NCT04535856	Active, not recruiting	I	NA	Therapeutic Study to Evaluate the Safety and Efficacy of DW-MSC in COVID-19 Patients	9	IV, low dose (50×10^6^ cells) High dose (1×10^6^ cells)	November 2020	March 2021	Indonesia
14	COVID-19	NCT04345601	Not yet recruiting	Early I	NA	Mesenchymal Stromal Cells for the Treatment of SARS-CoV-2 Induced Acute Respiratory Failure (COVID-19 Disease)	30	IV, 100×10^6^ MSCs	December 2020	September 2022	United States
15	COVID-19	NCT04461925	Recruiting	I-II	Placenta-MSCs	Treatment of Coronavirus COVID-19 Pneumonia (Pathogen SARS-CoV-2) With Cryopreserved Allogeneic P_MMSCs and UC-MMSCs	30	IV, 3 does of MSCs (1×10^6^ cells /kg BW at D1, D4, D7)	May 2020	December 2021	Ukraine
16	COVID-19	NCT04313322	Recruiting	I	WJ-MSCs	Treatment of COVID-19 Patients Using Wharton's Jelly-Mesenchymal Stem Cells	5	IV, 3 doses of 1×10^6^ cells/kg BW, 3 days apart form each other	March 2020	September 2020	Jordan
17	COVID-19	NCT04625738	Not yet recruiting	II	WJ-MSCs	Efficacy of Infusions of MSC From Wharton Jelly in the SARS-Cov-2 (COVID-19) Related Acute Respiratory Distress Syndrome	30	IV, D0: 1×10^6^ cells/kg BW; D3: 0.5×10^6^ cells/kg BW; D5: 0.5×10^6^ cells/kg BW	November 2020	August 2022	France
18	COVID-19	NCT04339660	Recruiting	I-II	UCMSCs	Clinical Research of Human Mesenchymal Stem Cells in the Treatment of COVID-19 Pneumonia	30	IV, 1×10^6^ cells/kg BW	April 2020	June 2020	China
19	COVID-19	NCT04273646	Not yet recruiting	NA	UCMSCs	Study of Human Umbilical Cord Mesenchymal Stem Cells in the Treatment of Severe COVID-19	48	IV, 4 does of MSCs (0.5×10^6^ cells/kg BW at Day 1, Day 3, Day 5, Day 7)	April 2020	February 2022	China
20	COVID-19	NCT04390139	Recruiting	I-II	WJ-MSCs	Efficacy and Safety Evaluation of Mesenchymal Stem Cells for the Treatment of Patients With Respiratory Distress Due to COVID-19	30	IV, 1×10^6^ cells/kg BW per dose at D1 and D3	May 2020	December 2020	Spain
21	COVID-19	NCT04457609	Recruiting	I	UCMSCs	Administration of Allogenic UC-MSCs as Adjuvant Therapy for Critically-Ill COVID-19 Patients	40	IV, 1×10^6^ cells/kg BW	July 2020	September 2020	Indonesia
22	COVID-19	NCT04452097	Not yet recruiting	I-II	UCMSCs	Use of hUC-MSC Product (BX-U001) for the Treatment of COVID-19 With ARDS	39	IV, low dose (0.5×10^6^ cells/kg BW); Middle dose (1×10^6^ cells/kg BW) high dose (1×10^6^ cells/kg BW)	February 2021	December 2021	NA
23	COVID-19	NCT04490486	Not yet recruiting	I	UCMSCs	Umbilical Cord Tissue (UC) Derived Mesenchymal Stem Cells (MSCs) Versus Placebo to Treat Acute Pulmonary Inflammation Due to COVID-19	21	IV, 100×10^6^ cells	July 2020	June 2024	United States
24	COVID-19	NCT03042143	Recruiting	I-II	UCMSCs	Repair of Acute Respiratory Distress Syndrome by Stromal Cell Administration (REALIST) (COVID-19)	75	IV, 100×10^6^cells; 200×10^6^cells; 400×10^6^ cells	January 2020	October 2022	United Kingdom
25	COVID-19	NCT04494386	Recruiting	I-II	UCMSCs	Umbilical Cord Lining Stem Cells (ULSC) in Patients With COVID-19 ARDS	60	IV, 100×10^6^cells per dose	July 2020	November 2021	United States
26		NCT04429763	Not yet recruiting	II	UCMSCs	Safety and Efficacy of Mesenchymal Stem Cells in the Management of Severe COVID-19 Pneumonia	30	IV, one dose of 1×10^6^ cells/kg BW	July 2020	November 2020	
27	COVID-19	NCT04565665	Recruiting	I	UCMSCs	Cord Blood-Derived Mesenchymal Stem Cells for the Treatment of COVID-19 Related Acute Respiratory Distress Syndrome	70	IV	July 2020	April 2021	United States
28	COVID-19	NCT04269525	Recruiting	II	UCMSCs	Umbilical Cord(UC)-Derived Mesenchymal Stem Cells(MSCs) Treatment for the 2019-novel Coronavirus(nCOV) Pneumonia	16	IV, 4 doses of MSCs (100×10^6^ cells/time at D1, D3, D5, D7)	February 2020	December 2020	China
29	COVID-19	NCT04333368	Active, not recruiting	I-II	UCMSCs	Cell Therapy Using Umbilical Cord-derived Mesenchymal Stromal Cells in SARS-CoV-2-related ARDS	47	IV, 1×10^6^ cells/kg BW at D1, D3, D5	April 2020	April 2022	France
30	COVID-19	NCT04390152	Recruiting	I-II	UCMSCs	Safety and Efficacy of Intravenous Wharton's Jelly Derived Mesenchymal Stem Cells in Acute Respiratory Distress Syndrome Due to COVID 19	40	IV, two doses of MSCs (50×10^6^ cells per dose)	May 2020	April 2022	Colombia
31	COVID-19	NCT04456361	Active, not recruiting	Early I	UCMSCs	Use of Mesenchymal Stem Cells in Acute Respiratory Distress Syndrome Caused by COVID-19	9	IV, 100×10^6^ cells	July 2020	December 2020	Mexico
32	COVID-19	NCT04399889	Recruiting	I-II	UCMSCs	hCT-MSCs for COVID19 ARDS	30	IV, 1×10^6^ cells/kg BW (max 100 million cells)	June 2020	July 2021	United States
33	COVID-19	NCT04398303	Not yet recruiting	I-II	UCMSCs	ACT-20 in Patients With Severe COVID-19 Pneumonia	70	IV, 1×10^6^ cells/kg BW	May 2020	October 2020	NA
34	COVID-19	NCT04392778	Recruiting	I-II	UCMSCs	Clinical Use of Stem Cells for the Treatment of Covid-19	30	IV, 3 dose of MSCs (3×10^6^ cells/kg BW at D1, D3, D6 )	April 2020	September 2020	Turkey
35	COVID-19	NCT04371601	Active, not recruiting	Early I	UCMSCs	Safety and Effectiveness of Mesenchymal Stem Cells in the Treatment of Pneumonia of Coronavirus Disease 2019	60	IV, 4 doses of MSCs (1×10^6^ cells/kg BW once every 4 days)	March 2020	December 2022	China
36	COVID-19	NCT04416139	Recruiting	II	UCMSCs	Mesenchymal Stem Cell for Acute Respiratory Distress Syndrome Due for COVID-19	10	IV, single dose of 1×10^6^ cells/kg BW	May 2020	May 2021	Mexico
37	COVID-19	NCT04397796	Recruiting	I	BMSCs	Study of the Safety of Therapeutic Tx with Immunomodulatory MSC in Adults With COVID-19 Infection Requiring Mechanical Ventilation	45	NA	August 2020	June 2021	US
38	COVID-19	NCT04346368	Not yet recruiting	I-II	BMSCs	Bone Marrow-Derived Mesenchymal Stem Cell Treatment for Severe Patients With Coronavirus Disease 2019 (COVID-19)	20	IV, 1×10^6^ cells/kg BW at D1	April 2020	December 2020	China
39	COVID-19	NCT04397471	Not yet recruiting	NA	BMSCs	A Study to Collect Bone Marrow for Process Development and Production of BM-MSC to Treat Severe COVID19 Pneumonitis	10	NA	May 2020	December 2021	United Kingdom
40	COVID-19	NCT04444271	Recruiting	II	BMSCs	Mesenchymal Stem Cell Infusion for COVID-19 Infection	20	IV, 2×10^6^ cells/kg BW at D1, D7	May 2020	September 2020	Pakistan
41	COVID-19	NCT04377334	Not yet recruiting	II	BMSCs	Mesenchymal Stem Cells (MSCs) in Inflammation-Resolution Programs of Coronavirus Disease 2019 (COVID-19) Induced Acute Respiratory Distress Syndrome (ARDS)	40	IV	October 2020	July 2021	Germany
42	COVID-19	NCT04400032	Recruiting	I	BMSCs	Cellular Immuno-Therapy for COVID-19 Acute Respiratory Distress Syndrome - Vanguard	9	IV, 75×10^6^ cells; 150×10^6^ cells; 270×10^6^ cells	May 2020	June 2021	Canada
43	COVID-19	NCT04445454	Recruiting	I-II	BMSCs	Mesenchymal Stromal Cell Therapy for Severe Covid-19 Infection	20	IV, 3 dose of 1.5-3.0×10^6^ cells/kg BW	June 2020	September 2022	Belgium
44	COVID-19	NCT04447833	Recruiting	I	BMSCs	Mesenchymal Stromal Cell Therapy For The Treatment Of Acute Respiratory Distress Syndrome	9	IV, group1: 1×10^6^ cells/kg BW; group2: 2×10^6^ cells/kg BW	June 2020	June 2025	Sweden
45	COVID-19	NCT04527224	Not yet recruiting	I-II	ADSCs	Study to Evaluate the Efficacy and Safety of AstroStem-V in Treatment of COVID-19 Pneumonia	10	NA	December 2020	April 2022	NA
46	COVID-19	NCT04522986	Not yet recruiting	I	ADSCs	An Exploratory Study of ADR-001 in Patients With Severe Pneumonia Caused by SARS-CoV-2 Infection	6	IV, 100×10^6^ cells once a week, total four times.	September 2020	December 2021	Japan
47	COVID-19	NCT04348461	Not yet recruiting	II	ADSCs	BAttLe Against COVID-19 Using MesenchYmal Stromal Cells	100	IV, two serial doses of 1.5 ×10^6^ cells/kg BW	April 2020	September 2020	Spain
48	COVID-19	NCT04352803	Not yet recruiting	I	Autologous ADSCs	Adipose Mesenchymal Cells for Abatement of SARS-CoV-2 Respiratory Compromise in COVID-19 Disease	20	IV, 0.5×10^6^ cells/kg BW	April 2020	April 2026	NA
49	COVID-19	NCT04366323	Active, not recruiting	I-II	ADSCs	Clinical Trial to Assess the Safety and Efficacy of Intravenous Administration of Allogeneic Adult Mesenchymal Stem Cells of Expanded Adipose Tissue in Patients With Severe Pneumonia Due to COVID-19	26	IV, two doses of 80 ×10^6^ cells	April 2020	October 2021	Spain
50	COVID-19	NCT04611256	Recruiting	I	ADSCs	Mesenchymal Stem Cells in Patients Diagnosed With COVID-19	20	IV, two doses of 1×10^6^ cells/kg BW at D1 and D3	August 2020	December 2020	Mexico
51	COVID-19	NCT04382547	Enrolling by invitation	I-II	Olfactory mucosa-derived MSCs	Treatment of Covid-19 Associated Pneumonia With Allogenic Pooled Olfactory Mucosa-derived Mesenchymal Stem Cells	40	NA	May 2020	June 2021	Belarus
52	COVID-19	NCT04302519	Not yet recruiting	Early I	Dental pulp-MSCs	Novel Coronavirus Induced Severe Pneumonia Treated by Dental Pulp Mesenchymal Stem Cells	24	IV, 1.0×10^6^ cells/kg BW at D1, D3 and D7	March 2020	July 2021	China
53	severe influenza pneumonia	NCT04282928	Not yet recruiting	I	UCMSCs	Efficacy and Safety of Umbilical Cord Mesenchymal Stem Cells for the Treatment of Severe Viral Pneumonian	40	IV, 1×10^6^ cells/kg BW	February 2020	March 2021	China
54	CABP	NCT03158727	Active, not recruiting	I/II	allogeneic ADSCs	Cx611-0204 SEPCELL Study (SEPCELL)	84	IV, 160×10^6^ cells on day 1 and day 3	January 2017	December 2021	France
55	ARDS	NCT03608592	Recruiting	NA	UCMSCs	Human Umbilical Cord Mesenchymal Stem Cells (MSCs) Therapy in ARDS	26	IV, 60×10^6^ cells in 100ml and infused in 2 hours	June 2018	December 2020	China
56	ARDS	NCT04289194	Recruiting	I-II	allogeneic ADSCs	Clinical Study to Assess the Safety and Preliminary Efficacy of HCR040 in Acute Respiratory Distress Syndrome	26	IV, Dose1: 1×10^6^ cells/kg BW; Dose 2: 2×10^6^ cells/kg BW	December 2019	July 2022	Spain
57	ARDS	NCT04347967	Not yet recruiting	I	UCMSCs	Mesenchymal Stem Cells for The Treatment of Acute Respiratory Distress Syndrome (ARDS)	18	NA	September 2020	December 2022	Taiwan,China
58	ARDS	NCT04602104	Not yet recruiting	I-II	MSCs-dervied exosomes	A Clinical Study of Mesenchymal Stem Cell Exosomes Nebulizer for the Treatment of ARDS	169	Aerosol inhalation, low-dose group: 2.0×10^8^ particles/day, one week; medium-dose group: 8.0×10^8^ particles/day, one week; high-dose group: 16.0×10^8^ particles/day, one week.(Phase Ӏ)	October 2020	June 2022	China
59	COPD	NCT04433104	Recruiting	I-II	UCMSCs	Umbilical Cord Mesenchymal Stem Cells Transplantation in the Treatment of Chronic Obstructive Pulmonary Disease	40	IV, 1×10^6^ cells /kg BW	June 2020	February 2022	Vietnam
60	COPD	NCT04047810	Recruiting	I	NA	Mesenchymal Stem Cells in the Treatment of Subjects With Advance Chronic Obstructive Pulmonary Disease (COPD)	15	IV, 0.5-2×10^6^ cells /kg BW	January 2020	August 2021	US
61	COPD	NCT04206007	Recruiting	I	UCMSCs	Mesenchymal Stem Cells for The Treatment of Chronic Obstructive Pulmonary Disease	9	IV	June 2020	December 2022	Taiwan, China
62	COPD	NCT04018729	Not yet recruiting	II-III	Allogenic BMSCs	Cell Therapy Associated With Endobronchial Valve	34	Bronchial injection	November 2019	February 2021	NA
63	COPD	NCT03909750	Recruiting	I	Autologous ADSCs	Use of Autologous Stem/Stromal Cells In Chronic Lung Disorders: Obstructive (COPD) & Restrictive (RLD)	50	IV	April 2019	September 2025	US
64	COPD	NCT02946658	Active, not recruiting	I-II	Autologous ADSCs	Use of Autologous, Adult Adipose-Derived Stem/Stromal Cells In Chronic Lung Disorders	100	IV	October 2016	March 2023	US
65	BPD	NCT03558334	Recruiting	I	UCMSCs	Human Mesenchymal Stem Cells For Bronchopulmonary Dysplasia	12	IV, Dose A: 1× 10^6^ cells/kg BW; Dose B: 5×10^6^ cells/kg BW	June 2018	June 2022	China
66	BPD	NCT03873506	Recruiting	NA	NA	Follow-Up Study of Mesenchymal Stem Cells for Bronchopulmonary Dysplasia (NCT03558334 )	30	NA	July 2018	December 2020	China
67	BPD	NCT03774537	Recruiting	I-II	UCMSCs	Human Mesenchymal Stem Cells For Infants At High Risk For Bronchopulmonary Dysplasia	20	IV, Dose A:1× 10^6^ cells/kg BW; Dose B: 5×10^6^ cells/kg BW	March 2019	December 2021	China
68	BPD	NCT03392467	Recruiting	II	UCMSCs	PNEUMOSTEM for the Prevention and Treatment of Severe BPD in Premature Infants	60	Intratracheal, 1.0×10^7^ cells/kg BW	August 2018	July 2021	Korea
69	BPD	NCT04003857	Recruiting	II	NA	Follow-up Study of Safety and Efficacy in Subjects Who Completed PNEUMOSTEM® Phase II (MP-CR-012) Clinical Trial (NCT03392467)	60	NA	July 2019	June 2027	Korea
70	BPD	NCT04255147	Not yet recruiting	I	UCMSCs	Cellular Therapy for Extreme Preterm Infants at Risk of Developing Bronchopulmonary Dysplasia	9	IV, Group 1: 1× 10^6^ cells/kg BW (3 patients); Group 2: 3× 10^6^ cells/kg BW (3 patients); Group 3: 10×10^6^ cells/kg BW (3 patients)	February 2020	December 2035	Canada
71	BPD	NCT02443961	Recruiting	I	NA	Mesenchymal Stem Cell Therapy for Bronchopulmonary Dysplasia in Preterm Babies	10	NA	April 2019	April 2025	Spain
72	BPD	NCT03378063	Recruiting	Early I	UCMSCs	Stem Cells for Bronchopulmonary Dysplasia	100	NA	November 2017	December 2022	China
73	BPD	NCT03601416	Not yet recruiting	II	UCMSCs	Human Mesenchymal Stem Cells For Moderate and Severe Bronchopulmonary Dysplasia	57	IV, Dose A: 1×10^6^ cells/kg BW; Dose B: 5×10^6^ cells/kg BW	July 2019	December 2021	China
74	BPD	NCT03645525	Recruiting	I-II	UCMSCs	Intratracheal Umbilical Cord-derived Mesenchymal Stem Cell for the Treatment of Bronchopulmonary Dysplasia (BPD)	180	Intratracheal instillate, 2×10^7^ cells/kg BW once	December 2019	October 2020	China
75	BPD	NCT03631420	Recruiting	I	UCMSCs	Mesenchymal Stem Cells for The Treatment of Bronchopulmonary Dysplasia in Infants	9	Intratracheal instillate, group 1: 3×10^6^ cells/kg BW; group 2: 10×10^6^ cells/kg BW; group 3: 30×10^6^ cells/kg BW	October 2018	October 2022	Taiwan, China
76	BPD	NCT04062136	Recruiting	I	UCMSC	Umbilical Cord Mesenchymal Stem Cells Transplantation in the Treatment of Bronchopulmonary Dysplasia	10	IV, twice of 1 ×10^6^ cells/kg BW, one week apart	March 2019	November 2020	Vietnam
77	BPD	NCT03857841	Recruiting	I	BMSCs-derived exosomes	A Safety Study of IV Stem Cell-derived Extracellular Vesicles (UNEX-42) in Preterm Neonates at High Risk for BPD	18	IV	June 2019	December 2021	US
78	CTD-ILD	NCT03929120	Recruiting	I	Allogeneic BMSCs	Allogeneic Bone Marrow Mesenchymal Stem Cells for Patients With Interstitial Lung Disease (ILD) & Connective Tissue Disorders (CTD)	10	IV, 0.5-1×10^6^ cells/kg BW	November 2019	December 2021	US
79	SSc-ILD	NCT04432545	Available	NA	Wharton's jelly-dervied MSCs	Infusion of Allogeneic Mesenchymal Stem Cells in Patients With Diffuse Cutaneous Systemic Sclerosis With Refractory Pulmonary Involvement	NA	IV, 2×10^6^ cells/kg BW	June 2020	NA	Colombia
80	Lung cancer	NCT03298763	Recruiting	I	MSCs-TRAIL	Targeted Stem Cells Expressing TRAIL as a Therapy for Lung Cancer (TACTICAL)	46	IV, 4×10^8^ cells	March 2019	September 2025	United Kingdom
81	PAH	NCT04055415	Recruiting	I	allogeneic ADSCs	Clinical Study of Adipose Derived Mesenchymal Stem Cells for Treatment of Pulmonary Arterial Hypertension	60	IV, 1×10^6^ cells/kg BW	August 2019	February 2021	China

NA, not applicable; MSCs, mesenchymal stem cells; IV, intravenously; BW, body weight; D, day; WJ-MSCs, Wharton's Jelly-derived MSCs; UCMSCs, umbilical cord-derived MSCs; BMSCs, bone marrow-derived MSCs; ADSCs, adipose tissue derived-MSCs. COPD, chronic obstructive pulmonary disease; BPD, bronchopulmonary dysplasia; ILD, interstitial lung diseases; CTD, connective tissue disease; SSc, systemic sclerosis; TRAIL, tumour necrosis factor (TNF)-related apoptosis inducing ligand; PAH, pulmonary arterial hypertension.

## References

[1] Li H, Liu SM, Yu XH, Tang SL, Tang CK (2020). Coronavirus disease 2019 (COVID-19): current status and future perspectives. Int J Antimicrob Agents.

[2] Zhang N, Zhang H, Tang Y, Zhang H, Ma A, Xu F (2021). Risk factors for illness severity in patients with COVID-19 pneumonia: a prospective cohort study. Int J Med Sci.

[3] Liao SX, Sun PP, Gu YH, Rao XM, Zhang LY, Ou-Yang Y (2019). Autophagy and pulmonary disease. Ther Adv Respir Dis.

[4] Young KA, Dilling DF (2019). The Future of Lung Transplantation. Chest.

[5] Lou S, Duan Y, Nie H, Cui X, Du J, Yao Y Mesenchymal stem cells: Biological characteristics and application in disease therapy. Biochimie. 2021: S0300-9084(21)00073-0.

[6] Deng L, Li H, Su X, Zhang Y, Xu H, Fan L (2020). Chlorzoxazone, a small molecule drug, augments immunosuppressive capacity of mesenchymal stem cells via modulation of FOXO3 phosphorylation. Cell Death Dis.

[7] Jiang W, Xu J (2020). Immune modulation by mesenchymal stem cells. Cell Prolif.

[8] Brown C, McKee C, Bakshi S, Walker K, Hakman E, Halassy S (2019). Mesenchymal stem cells: Cell therapy and regeneration potential. J Tissue Eng Regen Med.

[9] Yun CW, Lee SH (2019). Potential and Therapeutic Efficacy of Cell-based Therapy Using Mesenchymal Stem Cells for Acute/chronic Kidney Disease. Int J Mol Sci.

[10] Premer C, Schulman IH, Jackson JS The role of mesenchymal stem/stromal cells in the acute clinical setting. Am J Emerg Med. 2020: S0735-6757(20)31044-5.

[11] Friedenstein AJ, Chailakhjan RK, Lalykina KS (1970). The development of fibroblast colonies in monolayer cultures of guinea-pig bonemarrow and spleen cells. Cell Tissue Kinet.

[12] Nehlin JO, Jafari A, Tencerova M, Kassem M (2019). Aging and lineage allocation changes of bone marrow skeletal (stromal) stemcells. Bone.

[13] Ding DC, Chang YH, Shyu WC, Lin SZ (2015). Human umbilical cord mesenchymal stem cells: a new era for stem cell therapy. Cell Transplant.

[14] Seo Y, Shin TH, Kim HS (2019). Current Strategies to Enhance Adipose Stem Cell Function: An Update. Int J Mol Sci.

[15] Kuca-Warnawin E, Skalska U, Janicka I, Musiałowicz U, Bonek K, Głuszko P (2019). The Phenotype and Secretory Activity of Adipose-Derived Mesenchymal Stem Cells (ASCs) of Patients with Rheumatic Diseases. Cells.

[16] Worthington EN, Hagood JS (2020). Therapeutic Use of Extracellular Vesicles for Acute and Chronic Lung Disease. Int J Mol Sci.

[17] Harrell CR, Jovicic N, Djonov V, Arsenijevic N, Volarevic V (2019). Mesenchymal Stem Cell-Derived Exosomes and Other Extracellular Vesicles as New Remedies in the Therapy of Inflammatory Diseases. Cells.

[18] Abumaree MH, Abomaray FM, Alshehri NA, Almutairi A, AlAskar AS, Kalionis B (2016). Phenotypic and Functional Characterization of Mesenchymal Stem/Multipotent Stromal Cells From Decidua Parietalis of Human Term Placenta. Reprod Sci.

[19] Bae S, Shim SH, Park CW, Son HK, Lee HJ, Son JY (2011). Combined omics analysis identifies transmembrane 4 L6 family member 1 as a surface protein marker specific to human mesenchymal stem cells. Stem Cells Dev.

[20] Waterman RS, Tomchuck SL, Henkle SL, Betancourt AM (2010). A new mesenchymal stem cell (MSC) paradigm: polarization into a pro-inflammatory MSC1 or an Immunosuppressive MSC2 phenotype. PLoS One.

[21] Dabrowska S, Andrzejewska A, Janowski M, Lukomska B (2021). Immunomodulatory and Regenerative Effects of Mesenchymal Stem Cells and Extracellular Vesicles: Therapeutic Outlook for Inflammatory and Degenerative Diseases. Front Immunol.

[22] Shi Y, Wang Y, Li Q, Liu K, Hou J, Shao C (2018). Immunoregulatory mechanisms of mesenchymal stem and stromal cells in inflammatory diseases. Nat Rev Nephrol.

[23] Jiang W, Xu J (2020). Immune modulation by mesenchymal stem cells. Cell Prolif.

[24] Melenotte C, Silvin A, Goubet AG, Lahmar I, Dubuisson A, Zumla A (2020). Immune responses during COVID-19 infection. Oncoimmunology.

[25] Fu X, Liu G, Halim A, Ju Y, Luo Q, Song AG (2019). Mesenchymal Stem Cell Migration and Tissue Repair. Cells.

[26] Szydlak R (2019). Mesenchymal stem cells' homing and cardiac tissue repair. Acta Biochim Pol.

[27] Hu L, Yin C, Zhao F, Ali A, Ma J, Qian A (2018). Mesenchymal Stem Cells: Cell Fate Decision to Osteoblast or Adipocyte and Application in Osteoporosis Treatment. Int J Mol Sci.

[28] Qian Q, Qian H, Zhang X, Zhu W, Yan Y, Ye S (2012). 5-Azacytidine induces cardiac differentiation of human umbilical cord-derived mesenchymal stem cells by activating extracellular regulated kinase. Stem Cells Dev.

[29] Zhang C, Lin Y, Liu Q, He J, Xiang P, Wang D (2020). Growth differentiation factor 11 promotes differentiation of MSCs into endothelial-like cells for angiogenesis. Cell Mol Med.

[30] Sid-Otmane C, Perrault LP, Ly HQ (2020). Mesenchymal stem cell mediates cardiac repair through autocrine, paracrine and endocrine axes. J Transl Med.

[31] Lee BC, Kim HS, Shin TH, Kang I, Lee JY, Kim JJ (2016). PGE2 maintains self-renewal of human adult stem cells via EP2-mediated autocrine signaling and its production is regulated by cell-to-cell contact. Sci Rep.

[32] Watt SM, Gullo F, van der Garde M, Markeson D, Camicia R, Khoo CP (2013). The angiogenic properties of mesenchymal stem/stromal cells and their therapeutic potential. Br Med Bull.

[33] Roura S, Bagó JR, Soler-Botija C, Pujal JM, Gálvez-Montón C, Prat-Vidal C (2012). Human umbilical cord blood-derived mesenchymal stem cells promote vasculargrowth *in vivo*. PLoS One.

[34] Gong M, Yu B, Wang J, Wang Y, Liu M, Paul C (2017). Mesenchymal stem cells release exosomes that transfer miRNAs to endothelial cells and promote angiogenesis. Oncotarget.

[35] Lu GD, Cheng P, Liu T, Wang Z (2020). BMSC-Derived Exosomal miR-29a Promotes Angiogenesis and Osteogenesis. Front Cell Dev Biol.

[36] Khan A, Mann L, Papanna R, Lyu MA, Singh CR, Olson S (2017). Mesenchymal stem cells internalize Mycobacterium tuberculosis through scavenger receptors and restrict bacterial growth through autophagy. Sci Rep.

[37] Marx C, Gardner S, Harman RM, Van de Walle GR (2020). The mesenchymal stromal cell secretome impairs methicillin-resistantStaphylococcus aureus biofilms via cysteine protease activity in the equinemodel. Stem Cells Transl Med.

[38] Russell KA, Garbin LC, Wong JM, Koch TG (2020). Mesenchymal Stromal Cells as Potential Antimicrobial for Veterinary Use-A Comprehensive Review. Front Microbiol.

[39] Morrison TJ, Jackson MV, Cunningham EK, Kissenpfennig A, McAuley DF, O'Kane CM (2017). Mesenchymal Stromal Cells Modulate Macrophages in Clinically Relevant LungInjury Models by Extracellular Vesicle Mitochondrial Transfer. Am J Respir Crit Care Med.

[40] Chen R, Xie Y, Zhong X, Chen F, Gong Y, Wang N (2021). MSCs derived from amniotic fluid and umbilical cord require different administration schemes and exert different curative effects on different tissues in rats with CLP-induced sepsis. Stem Cell Res Ther.

[41] Hong J, Hueckelhoven A, Wang L, Schmitt A, Wuchter P, Tabarkiewicz J (2016). Indoleamine 2,3-dioxygenase mediates inhibition of virus-specific CD8(+) T cell proliferation by human mesenchymal stromal cells. Cytotherapy.

[42] Gholizadeh-Ghaleh Aziz S, Alipour S, Ranjbarvan P, Azari A, Babaei G, Golchin A (2021). Critical roles of TLRs on the polarization of mesenchymal stem cells for cell therapy of viral infections: a notice for COVID-19 treatment. Comp Clin Path.

[43] Liu T, Zhang Y, Shen Z, Zou X, Chen X, Chen L (2017). Immunomodulatory effects of OX40Ig gene-modified adipose tissue-derived mesenchymal stem cells on rat kidney transplantation. Int J Mol Med.

[44] Geng J, Liu T, Jiang J, Li G, Huang ZW, Wang YL (2020). The expression ICOSIg gene modified by eukaryotic expression vector in rat adipose tissue-derived mesenchymal stem cells. Tianjin Med J.

[45] Damasceno PKF, de Santana TA, Santos GC, Orge ID, Silva DN, Albuquerque JF (2020). Genetic Engineering as a Strategy to Improve the Therapeutic Efficacy of Mesenchymal Stem/Stromal Cells in Regenerative Medicine. Front Cell Dev Biol.

[46] Janik E, Niemcewicz M, Ceremuga M, Krzowski L, Saluk-Bijak J, Bijak M (2020). Various Aspects of a Gene Editing System-CRISPR-Cas9. Int J Mol Sci.

[47] Varkouhi AK, Monteiro APT, Tsoporis JN, Mei SHJ, Stewart DJ, Dos Santos CC (2020). Genetically Modified Mesenchymal Stromal/Stem Cells: Application in Critical Illness. Stem Cell Rev Rep.

[48] Weiss DJ, Casaburi R, Flannery R, LeRoux-Williams M, Tashkin DP (2013). A placebo-controlled, randomized trial of mesenchymal stem cells in COPD. Chest.

[49] ARDS Definition Task Force, Ranieri VM, Rubenfeld GD, Thompson BT, Ferguson ND, Caldwell E (2012). Acute respiratory distress syndrome: the Berlin Definition. JAMA.

[50] Yadav H, Thompson BT, Gajic O (2017). Fifty Years of Research in ARDS. Is Acute Respiratory Distress Syndrome a Preventable Disease?. Am J Respir Crit Care Med.

[51] Silva PL, Pelosi P, Rocco PRM (2020). Personalized pharmacological therapy for ARDS: a light at the end of the tunnel. Expert Opin Investig Drugs.

[52] Zheng G, Huang L, Tong H, Shu Q, Hu Y, Ge M (2014). Treatment of acute respiratory distress syndrome with allogeneic adipose-derived mesenchymal stem cells: a randomized, placebo-controlled pilot study. Respir Res.

[53] Wilson JG, Liu KD, Zhuo H, Caballero L, McMillan M, Fang X (2015). Mesenchymal stem (stromal) cells for treatment of ARDS: a phase 1 clinical trial. Lancet Respir Med.

[54] Chen J, Hu C, Chen L, Tang L, Zhu Y, Xu X (2020). Clinical Study of Mesenchymal Stem Cell Treatment for Acute Respiratory Distress Syndrome Induced by Epidemic Influenza A (H7N9) Infection: A Hint for COVID-19 Treatment. Engineering (Beijing).

[55] Wu Z, McGoogan JM (2020). Characteristics of and Important Lessons From the Coronavirus Disease 2019 (COVID-19) Outbreak in China: Summary of a Report of 72 314 Cases From the Chinese Center for Disease Control and Prevention. JAMA.

[56] Golchin A, Seyedjafari E, Ardeshirylajimi A (2020). Mesenchymal Stem Cell Therapy for COVID-19: Present or Future. Stem Cell Rev Rep.

[57] Leng Z, Zhu R, Hou W, Feng Y, Yang Y, Han Q (2020). Transplantation of ACE2- Mesenchymal Stem Cells Improves the Outcome of Patients with COVID-19 Pneumonia. Aging Dis.

[58] Liang B, Chen J, Li T, Wu H, Yang W, Li Y (2020). Clinical remission of a critically ill COVID-19 patient treated by human umbilical cord mesenchymal stem cells: A case report. Medicine.

[59] Zhang Y, Ding J, Ren S, Wang W, Yang Y, Li S (2020). Intravenous infusion of human umbilical cord Wharton's jelly-derived mesenchymal stem cells as a potential treatment for patients with COVID-19 pneumonia. Stem Cell Res Ther.

[60] Ruijin Hospital. A Pilot Clinical Study on Aerosol Inhalation of the Exosomes Derived From Allogenic Adipose Mesenchymal Stem Cells in the Treatment of Severe Patients With Novel Coronavirus Pneumonia.

[61] State-Financed Health Facility. The Protocol of Evaluation of Safety and Efficiency of Method of Exosome Inhalation in SARS-CoV-2 Associated Two-Sided Pneumonia.

[62] ClinicalTrials.gov Mesenchymal Stem Cell Treatment for Pneumonia Patients Infected With 2019 Novel Coronavirus. ClinicalTrials.gov Identifier: NCT04252118. Accessed date 3/15/2020. https://clinicaltrials.gov/ct2/show/ NCT04252118.

[63] ClinicalTrials.gov Umbilical Cord (UC)-Derived Mesenchymal Stem Cells (MSCs) Treatment for the 2019-novel Coronavirus (nCOV) Pneumonia. ClinicalTrials.gov Identifier: NCT04269525. Accessed date 3/15/2020. https://clinicaltrials.gov/ct2/show/NCT04269525?draw=2.

[64] Horcajada JP, Salata RA, Álvarez-Sala R, Nitu FM, Lawrence L, Quintas M (2019). A Phase 3 Study to Compare Delafloxacin With Moxifloxacin for the Treatment of Adults With Community-Acquired Bacterial Pneumonia (DEFINE-CABP). Open Forum Infect Dis.

[65] McCurdy S, Keedy K, Lawrence L, Nenninger A, Sheets A, Quintas M (2020). Efficacy of Delafloxacin versus Moxifloxacin against Bacterial Respiratory Pathogens in Adults with Community-Acquired Bacterial Pneumonia (CABP): Microbiology Results from the Delafloxacin Phase 3 CABP Trial. Antimicrob Agents Chemother.

[66] Laterre PF, Sánchez-García M, van der Poll T, de la Rosa O, Cadogan KA, Lombardo E (2020). A phase Ib/IIa, randomised, double-blind, multicentre trial to assess the safety and efficacy of expanded Cx611 allogeneic adipose-derived stem cells (eASCs) for the treatment of patients with community-acquired bacterial pneumonia admitted to the intensive care unit. BMC Pulm Med.

[67] Skrahin A, Jenkins HE, Hurevich H, Solodovnikova V, Isaikina Y, Klimuk D (2016). Effectiveness of a novel cellular therapy to treat multidrug-resistant tuberculosis. J Clin Tuberc Other Mycobact Dis.

[68] Skrahin A, Ahmed RK, Ferrara G, Rane L, Poiret T, Isaikina Y (2014). Autologous mesenchymal stromal cell infusion as adjunct treatment in patients with multidrug and extensively drug-resistant tuberculosis: an open-label phase 1 safety trial. Lancet Respir Med.

[69] Nellums LB, Rustage K, Hargreaves S, Friedland JS (2018). Multidrug-resistant tuberculosis treatment adherence in migrants: a systematic review and meta-analysis. BMC Med.

[70] Whittaker Brown SA, Braman S (2020). Recent Advances in the Management of Acute Exacerbations of Chronic Obstructive Pulmonary Disease. Med Clin North Am.

[71] Rabe KF, Watz H (2017). Chronic obstructive pulmonary disease. Lancet.

[72] Stolk J, Broekman W, Mauad T, Zwaginga JJ, Roelofs H, Fibbe WE (2016). A phase I study for intravenous autologous mesenchymal stromal cell administration to patients with severe emphysema. QJM.

[73] Namba F (2019). Mesenchymal stem cells for the prevention of bronchopulmonary dysplasia. Pediatr Int.

[74] Bonadies L, Zaramella P, Porzionato A, Perilongo G, Muraca M, Baraldi E (2020). Present and Future of Bronchopulmonary Dysplasia. J Clin Med.

[75] Jobe AH (2016). Mechanisms of Lung Injury and Bronchopulmonary Dysplasia. Am J Perinatol.

[76] Chang YS, Ahn SY, Yoo HS, Sung SI, Choi SJ, Oh WI (2014). Mesenchymal stem cells for bronchopulmonary dysplasia: phase 1 dose-escalation clinical trial. J Pediatr.

[77] Ahn SY, Chang YS, Kim JH, Sung SI, Park WS (2017). Two-Year Follow-Up Outcomes of Premature Infants Enrolled in the Phase I Trial of Mesenchymal Stem Cells Transplantation for Bronchopulmonary Dysplasia. J Pediatr.

[78] Medipost Co Ltd. A Multi-center, Randomized, Double-blind, Parallel, Placebo-controlled Phase II Clinical Trial to Evaluate the Efficacy and Safety of PNEUMOSTEM for the Prevention and Treatment of Severe Bronchopulmonary Dysplasia in Premature Infants.

[79] Medipost Co Ltd. Follow-up Study of Safety and Efficacy in Subjects Who Completed PNEUMOSTEM® Phase II (MP-CR-012) Clinical Trial.

[80] Wu X, Xia Y, Zhou O, Song Y, Zhang X, Tian D (2020). Allogeneic human umbilical cord-derived mesenchymal stem cells for severe bronchopulmonary dysplasia in children: study protocol for a randomized controlled trial (MSC-BPD trial). Trials.

[81] Saito S, Alkhatib A, Kolls JK, Kondoh Y, Lasky JA (2019). Pharmacotherapy and adjunctive treatment for idiopathic pulmonary fibrosis (IPF). J Thorac Dis.

[82] Siniscalco D, Sullo N, Maione S, Rossi F, D'Agostino B (2008). Stem cell therapy: the great promise in lung disease. Ther Adv Respir Dis.

[83] Li X, Yue S, Luo Z (2017). Mesenchymal stem cells in idiopathic pulmonary fibrosis. Oncotarget.

[84] Chambers DC, Enever D, Ilic N, Sparks L, Whitelaw K, Ayres J (2014). A phase 1b study of placenta-derived mesenchymal stromal cells in patients with idiopathic pulmonary fibrosis. Respirology.

[85] Averyanov A, Koroleva I, Konoplyannikov M, Revkova V, Lesnyak V, Kalsin V (2020). First-in-human high-cumulative-dose stem cell therapy in idiopathic pulmonary fibrosis with rapid lung function decline. Stem Cells Transl Med.

[86] Glassberg MK, Minkiewicz J, Toonkel RL, Simonet ES, Rubio GA, DiFede D (2017). Allogeneic Human Mesenchymal Stem Cells in Patients With Idiopathic Pulmonary Fibrosis via Intravenous Delivery (AETHER): A Phase I Safety Clinical Trial. Chest.

[87] Spagnolo P, Distler O, Ryerson CJ (2021). Mechanisms of progressive fibrosis in connective tissue disease (CTD)-associated interstitial lung diseases (ILDs). Ann Rheum Dis.

[88] Jee AS, Corte TJ (2019). Current and Emerging Drug Therapies for Connective Tissue Disease-Interstitial Lung Disease (CTD-ILD). Drugs.

[89] Barnes H, Holland AE, Westall GP, Goh NS, Glaspole IN (2018). Cyclophosphamide for connective tissue disease-associated interstitial lung disease. Cochrane Database Syst Rev.

[90] Maria AT, Maumus M, Le Quellec A, Jorgensen C, Noël D, Guilpain P (2017). Adipose-Derived Mesenchymal Stem Cells in Autoimmune Disorders: State of the Art and Perspectives for Systemic Sclerosis. Clin Rev Allergy Immunol.

[91] Mayo Clinic. A Phase I Study to Evaluate the Safety of Allogeneic Bone Marrow Derived Mesenchymal Stem Cells for Interstitial Lung Disease in patients with Connective Tissue Disorders.

[92] Universidad de la Sabana. Infusion of Allogeneic Stromal Mesenchymal Stem Cells From Wharton´s Jelly in Patients With Diffuse Cutaneous Systemic Sclerosis With Refractory Pulmonary Involvement to Treatment.

[93] Zhao H, Xie Y, Wang J, Li X, Li J (2019). Pulmonary rehabilitation for pneumoconiosis: protocol for a systematic review and meta-analysis. BMJ Open.

[94] Hall NB, Blackley DJ, Halldin CN, Laney AS (2019). Current Review of Pneumoconiosis Among US Coal Miners. Curr Environ Health Rep.

[95] Jianwu Dai. A Multicenter, Randomized, Single-blind, Parallel-group Study of Combined Large Volume WLL With Clinical Grade Umbilical Cord Mesenchymal Stem Cells(MSC) Transplantation for Treatment of Pneumoconiosis.

[96] He Y, Thummuri D, Zheng G, Okunieff P, Citrin DE, Vujaskovic Z (2019). Cellular senescence and radiation-induced pulmonary fibrosis. Transl Res.

[97] Jianwu Dai. Phase I Study of Radiation-induced Pulmonary Fibrosis Treated with Clinical Grade Umbilical Cord Mesenchymal Stem Cells.

[98] Kotecha S, Paraskeva MA, Levin K, Snell GI (2020). An update on chronic lung allograft dysfunction. Ann Transl Med.

[99] Williams KM (2017). How I treat bronchiolitis obliterans syndrome after hematopoietic stem cell transplantation. Blood.

[100] Chambers DC, Enever D, Lawrence S, Sturm MJ, Herrmann R, Yerkovich S (2017). Mesenchymal Stromal Cell Therapy for Chronic Lung Allograft Dysfunction: Results of a First-in-Man Study. Stem Cells Transl Med.

[101] Chen S, Zhao K, Lin R, Wang S, Fan Z, Huang F (2019). The efficacy of mesenchymal stem cells in bronchiolitis obliterans syndrome after allogeneic HSCT: A multicenter prospective cohort study. EBioMedicine.

[102] Fakiruddin KS, Ghazalli N, Lim MN, Zakaria Z, Abdullah S (2018). Mesenchymal Stem Cell Expressing TRAIL as Targeted Therapy against Sensitised Tumour. Int J Mol Sci.

[103] University College, London. Targeted Stem Cells Expressing TRAIL as a Therapy for Lung Cancer.

[104] Fakıoğlu DM, Altun B (2020). New Therapeutic Approaches in Cystic Fibrosis. Turk J Pharm Sci.

[105] Sutton MT, Fletcher D, Ghosh SK, Weinberg A, van Heeckeren R, Kaur S (2016). Antimicrobial Properties of Mesenchymal Stem Cells: Therapeutic Potential for Cystic Fibrosis Infection, and Treatment. Stem Cells Int.

[106] Erica Roesch. A Phase I, Single Center, Open Label, Single Dose, Dose Escalation Study Assessing the Safety and Tolerability of AllogeneiC MEsenchymAl Stem CEll Infusion in Adults With Cystic Fibrosis-CEASE CF.

[107] Antoniu SA (2018). Investigational inhaled therapies for non-CF bronchiectasis. Expert Opin Investig Drugs.

[108] Marilyn Glassberg. A Phase I, Trial to Evaluate the Safety, Tolerability, and Potential Efficacy of Allogeneic Human Mesenchymal Stem Cell (hMSC) Infusion in Patients With Non-Cystic Fibrosis Bronchiectasis.

[109] Pulido T, Adzerikho I, Channick RN, Delcroix M, Galiè N, Ghofrani HA (2013). Macitentan and morbidity and mortality in pulmonary arterial hypertension. N Engl J Med.

[110] Liaocheng People's Hospital. Safety and Efficacy of Transplantation of Adipose Derived Mesenchymal Stem Cells to Treat Pulmonary Arterial Hypertension.

[111] Malaviya R, Abramova EV, Rancourt RC, Sunil VR, Napierala M, Weinstock D (2020). Progressive Lung Injury, Inflammation, and Fibrosis in Rats Following Inhalation of Sulfur Mustard. Toxicol Sci.

[112] Ghazanfari T, Ghaffarpour S, Kariminia A, Salehi E, Hashemi SM, Ardestani SK (2019). Circulating mesenchymal stem cells in sulfur mustard-exposed patients with long-term pulmonary complications. Toxicol Lett.

[113] Nejad-Moghaddam A, Ajdari S, Tahmasbpour E, Goodarzi H, Panahi Y, Ghanei M (2017). Adipose-Derived Mesenchymal Stem Cells for Treatment of Airway Injuries in A Patient after Long-Term Exposure to Sulfur Mustard. Cell J.

[114] Qin H, Zhao A (2020). Mesenchymal stem cell therapy for acute respiratory distress syndrome: from basic to clinics. Protein Cell.

[115] Volarevic V, Markovic BS, Gazdic M, Volarevic A, Jovicic N, Arsenijevic N (2018). Ethical and Safety Issues of Stem Cell-Based Therapy. Int J Med Sci.

[116] Wang YL, Wang F, Geng J (2020). Cytokine and cytokine storm. Tianjin Med J.

[117] Sinclair KA, Yerkovich ST, Hopkins PM, Fieuw AM, Ford P, Powell JE (2021). The autotaxin-lysophosphatidic acid pathway mediates mesenchymal cell recruitment and fibrotic contraction in lung transplant fibrosis. J Heart Lung Transplant.

[118] Goldberg A, Mitchell K, Soans J, Kim L, Zaidi R (2017). The use of mesenchymal stem cells for cartilage repair and regeneration: a systematic review. J Orthop Surg Res.

[119] Song N, Wakimoto H, Rossignoli F, Bhere D, Ciccocioppo R, Chen KS (2021). Mesenchymal stem cell immunomodulation: In pursuit of controlling COVID-19 related cytokine storm. Stem Cells.

